# Hypermucoviscosity Regulator RmpD Interacts with Wzc and Controls Capsular Polysaccharide Chain Length

**DOI:** 10.1128/mbio.00800-23

**Published:** 2023-05-04

**Authors:** Olga G. Ovchinnikova, Logan P. Treat, Tanisha Teelucksingh, Bradley R. Clarke, Taryn A. Miner, Chris Whitfield, Kimberly A. Walker, Virginia L. Miller

**Affiliations:** a Department of Molecular and Cellular Biology, University of Guelph, Guelph, Ontario, Canada; b Department of Microbiology and Immunology, University of North Carolina School of Medicine, Chapel Hill, North Carolina, USA; c Department of Genetics, University of North Carolina School of Medicine, Chapel Hill, North Carolina, USA; New York University School of Medicine

**Keywords:** capsule, RmpD, Wzc, hypermucoviscosity, hypervirulent, *Klebsiella*, capsule chain length

## Abstract

Klebsiella pneumoniae is a leading cause of nosocomial infections, including pneumonia, bacteremia, and urinary tract infections. Treatment options are increasingly restricted by the high prevalence of resistance to frontline antibiotics, including carbapenems, and the recently identified plasmid-conferred colistin resistance. The classical pathotype (cKp) is responsible for most of the nosocomial infections observed globally, and these isolates are often multidrug resistant. The hypervirulent pathotype (hvKp) is a primary pathogen capable of causing community-acquired infections in immunocompetent hosts. The hypermucoviscosity (HMV) phenotype is strongly associated with the increased virulence of hvKp isolates. Recent studies demonstrated that HMV requires capsule (CPS) synthesis and the small protein RmpD but is not dependent on the increased amount of capsule associated with hvKp. Here, we identified the structure of the capsular and extracellular polysaccharide isolated from hvKp strain KPPR1S (serotype K2) with and without RmpD. We found that the polymer repeat unit structure is the same in both strains and that it is identical to the K2 capsule. However, the chain length of CPS produced by strains expressing *rmpD* demonstrates more uniform length. This property was reconstituted in CPS from Escherichia coli isolates that possess the same CPS biosynthesis pathway as K. pneumoniae but naturally lack *rmpD.* Furthermore, we demonstrate that RmpD binds Wzc, a conserved capsule biosynthesis protein required for CPS polymerization and export. Based on these observations, we present a model for how the interaction of RmpD with Wzc could impact CPS chain length and HMV.

## INTRODUCTION

Klebsiella pneumoniae is a Gram-negative bacterium with a remarkable ability to cause a wide range of human diseases. It is a leading cause of nosocomial infections, such as pneumonia, urinary tract infections, and bacteremia (1). The treatment of K. pneumoniae infections is complicated by the prevalence of antibiotic resistance (including extended-spectrum β-lactamases and carbapenemases) (2–4). The recent identification of plasmid-conferred resistance to colistin (5) further restricts treatment options.

Recent bioinformatic analyses of the sequenced genomes from a large collection of K. pneumoniae isolates showed that K. pneumoniae is an exceptionally diverse species with an average genome size of 5 to 6 Mbp (6). The core genome consists of ~1,700 genes, but the pangenome (core and accessory genes) is well over 100,000 genes (7). K. pneumoniae isolates typically produce a polysaccharide capsule (CPS) that is essential for virulence and is an established virulence factor of this species (8–10). The structures of K. pneumoniae CPSs are extremely diverse and give rise to more than 130 known and predicted capsule serotypes (K-types) identified to date (11–13).

Isolates of K. pneumoniae associated with infections in humans are classified broadly into two pathotypes. The classical pathotype (cKp) is considered an opportunistic pathogen and often is multidrug resistant (1, 6). This pathotype is responsible for most of the global nosocomial and long-term nursing care facility infections caused by K. pneumoniae. cKp strains have a wide range of K-types and produce other virulence factors, such as type I and type III fimbriae and siderophores (enterobactin, and yersiniabactin in some cKp) (1). The hypervirulent pathotype (hvKp) is considered a primary pathogen, as these strains can be community acquired and are able to cause disease in immunocompetent hosts (14). In contrast to cKp, the K-types found in hvKp isolates are quite limited, with a vast majority confined to serotypes K1 or K2 (15). HvKp isolates have properties not typically found in cKp strains, including the production of additional siderophores, such as aerobactin and salmochelin. HvKp isolates often produce more CPS than cKp isolates (referred to as hypercapsule) and have a hypermucoviscosity phenotype (HMV) that is determined by the formation of a “string” of at least 5 mm after touching a colony on an agar plate (16). Genes associated with these traits, including hypercapsule and HMV, are present in large virulence plasmids or chromosomal *ICE*Kp islands (17–22). As the genes conferring antibiotic resistance and hypervirulence are carried on mobile elements, it is no surprise that reports of K. pneumoniae displaying the convergent phenotypes of carbapenem resistance (CR) and hypervirulence are on the rise (23–25).

CPS is essential for the full virulence of both cKp and hvKp, and HMV is an important contributor to the hypervirulence of hvKp strains (1). The capsule produced by K. pneumoniae is synthesized via a Wzy-dependent pathway based on characteristic features of the biosynthesis and export machinery. Wzy-dependent export is classified as a “group 1 capsule” in Escherichia coli, where the K30 capsule has served as a paradigm for analyses of this capsule-assembly pathway (26, 27). The fundamental organization of group 1 capsule (*cps*) operons in K. pneumoniae and E. coli is conserved (28). The conserved organization of *cps* operons a lateral gene transfer of the loci between these species, resulting in instances of the *cps* operons sharing genetic organization and context in multiple species (28, 29). Each *cps* operon contains serotype-specific genes dedicated to the synthesis of a particular CPS repeat-unit structure, as well as conserved genes, such as *wzi*, *wza*, *wzb*, and *wzc*, encoding proteins participating in the assembly and export of CPS in all the different K-types.

One of the earliest indications that CPS and HMV in K. pneumoniae are somehow linked came from the identification of a transcriptional regulator of capsule (*cps*) genes, RmpA (17). Further studies were done using K. pneumoniae strain KPPR1S (serotype K2, HMV^Pos^) which carries *rmpA* on the chromosome (10, 30). These studies showed that *rmpA* is the first gene of an operon that consists of at least three genes (*rmpADC*) (31). A Δ*rmpA* mutant is HMV^Neg^, and while it produces less CPS (based on measurement of uronic acid [UA] content in the CPS) than the wild-type hvKp strain, it still produces more CPS than most cKp isolates (17, 31, 32). The *rmpC* gene also is predicted to encode a transcriptional regulator. An in-frame deletion mutant of *rmpC* (Δ*rmpC*) has a similar effect on *cps* gene expression and CPS production as the Δ*rmpA* mutant, but unlike the Δ*rmpA* mutant, the Δ*rmpC* mutant retains the wild-type HMV^Pos^ phenotype (31). The Δ*rmpC* mutation was the first to clearly separate the hypercapsule phenotype from the HMV^Pos^ phenotype. The Δ*rmpD* mutant is also HMV^Neg^, but *cps* genes are transcribed, and it produces CPS in amounts comparable to the wild-type strain (33). Furthermore, in a Δ*rmpADC* mutant, the expression of *rmpD* alone is sufficient to restore the HMV^Pos^ phenotype (33). Thus, *rmpD* was the first gene demonstrated to be required specifically for the HMV phenotype. This same study analyzed mutations in two *cps* genes required for CPS synthesis, namely, *wcaJ* and *manC*. Both mutations resulted in an HMV^Neg^ phenotype that could not be reversed by the expression of *rmpD*, indicating that the synthesis of CPS is necessary for the HMV^Pos^ phenotype (33). An independent study in which a transposon library was screened for mutations that impact CPS and/or HMV also found that CPS production and HMV were tightly linked (34). Together, these observations suggest that there is a tight linkage between the HMV phenotype and the production of the capsule. Yet it remains unclear whether CPS alone is sufficient to confer an HMV^Pos^ phenotype.

The HMV phenotype is clearly central to K. pneumoniae pathogenesis, but ambiguity concerning the identity of polymeric component(s) in HMV^Pos^ isolates and the relationship to CPS are major limitations in our understanding. To address these limitations, we sought to unequivocally establish the structure of the HMV polysaccharide and to identify the role of RmpD in producing this substance.

## RESULTS

### The HMV polysaccharide is capsule.

While several studies have suggested a strong link between HMV and the capsule (33–35) the precise composition of the polymeric substance underpinning the HMV phenotype has remained elusive. Thus, the initial objective of this study was to unequivocally determine the composition of the substance that confers HMV. CPS analysis can be complicated by contamination with other cell glycans, and in the case of K. pneumoniae, an association between CPS and components of lipopolysaccharide (LPS) has been reported (36, 37). Therefore, to simplify the analysis, *wecA* was deleted to eliminate the synthesis of the LPS O antigen in K. pneumoniae KPPR1S. The WecA enzyme catalyzes the first step in the production of the enterobacterial common antigen and O antigen polysaccharides but it plays no role in the biosynthesis of K. pneumoniae CPS (26, 38). KPPR1S Δ*wecA* remains HMV^Pos^ (33). The total extracellular polysaccharide fraction comprising a cell-free (CPS^SUP^) and cell-bound (CPS^CELL^) polymer was isolated from KPPR1S Δ*wecA* and the corresponding (HMV^Neg^) KPPR1S Δ*wecA*Δ*rmpD* mutant.

The viscosity of the polymer fractions, particularly from HMV^Pos^ strains, was not amenable to existing purification workflows, so a new scheme was developed (see Fig. S1A in the supplemental material). Contaminating LPS (lipid A + core oligosaccharide) is typically removed by ultracentrifugation, but this removal was not possible, due either to the high viscosity of the dissolved crude exopolysaccharide (EPS) samples and/or the previously reported ionic interactions between the EPS and LPS core (36). To remove LPS, crude CPS^CELL^ and CPS^SUP^ fractions were subjected to mild acid hydrolysis, which cleaves Kdo linkages between lipid A and the core oligosaccharide (39); the resulting lipid A precipitate was then removed by centrifugation, and the soluble core oligosaccharide was removed by dialysis. Glycosidic linkages involving hexoses and hexuronic acids (in CPS) are known to withstand hydrolysis with 1% AcOH, although SDS-PAGE analysis of the resulting AcOH-treated CPS samples still showed a small reduction in chain length compared with the starting material (Fig. S1B). The basis for this change is uncertain, but it did not compromise further chemical analysis.

10.1128/mbio.00800-23.1 FIG S1 Purification of capsule from HMV-positive Klebsiella. (A) Scheme for polysaccharide purification. (B) Purified polysaccharides were separated by SDS-PAGE and stained with alcian blue and then with silver, as described in the Methods and Materials. Download FIG S1, file, 6 MB. Copyright © 2023 Ovchinnikova et al. 2023 Ovchinnikova et al. https://creativecommons.org/licenses/by/4.0/ This content is distributed under the terms of the Creative Commons Attribution 4.0 International license.

**FIG 1:**
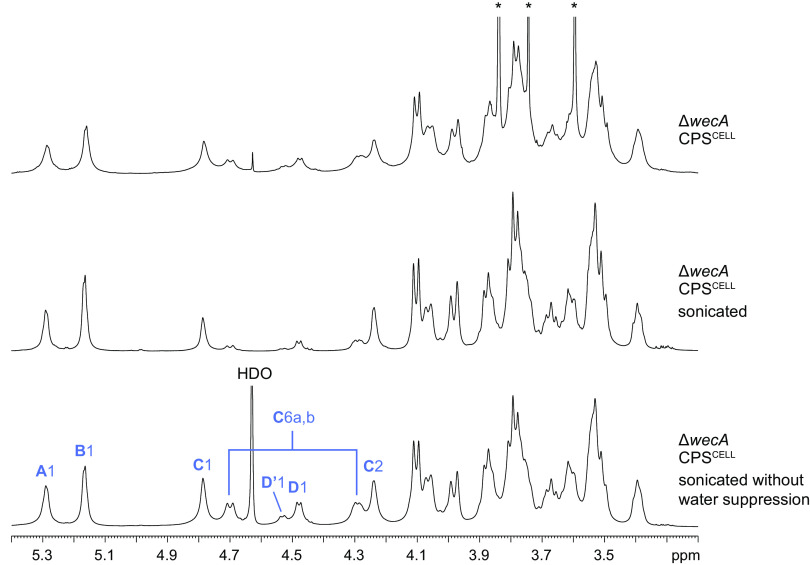
^1^H NMR spectra of AcOH-treated CPS^CELL^ from K. pneumoniae Δ*wecA.* The samples were analyzed before sonication and after sequential sonication and purification by gel permeation chromatography. The numbers refer to protons in sugar residues (denoted by blue capital letters) denoted in Table 1 and Fig. 2. Glc **D** linked to a non-*O*-acetylated Man residue is labeled as **D**’. The degree of *O*-acetylation was calculated by the integrated intensities of the signals for Man **C** H-1 at δ 4.79 (overlapping with non-*O*-acetylated Man H-1) and **C** H-6b at δ 4.70 in ^1^H NMR spectrum without water suppression in which the intensities of these signals are not altered. The asterisks mark signals from contaminating EDTA and Tris originating from the dialysis buffer.

The AcOH-treated CPS samples (~36 mg/mL) that were prepared for nuclear magnetic resonance (NMR) analyses were still viscous and, as expected, produced broad spectral lines in ^1^H NMR. To improve spectral resolution, the samples were sonicated in an ice/water bath and then were subjected to chromatography with Sephadex G-50 to remove residual buffer components remaining from the previous dialysis step. The viscosity of the CPS samples was reduced by this treatment, but all of it still eluted as relatively narrow peaks in the included volume of the column, demonstrating that the material was of high molecular weight. A comparison of ^1^H NMR spectra before and after sonication showed improved spectral resolution after this treatment (Fig. 1).

**FIG 2:**
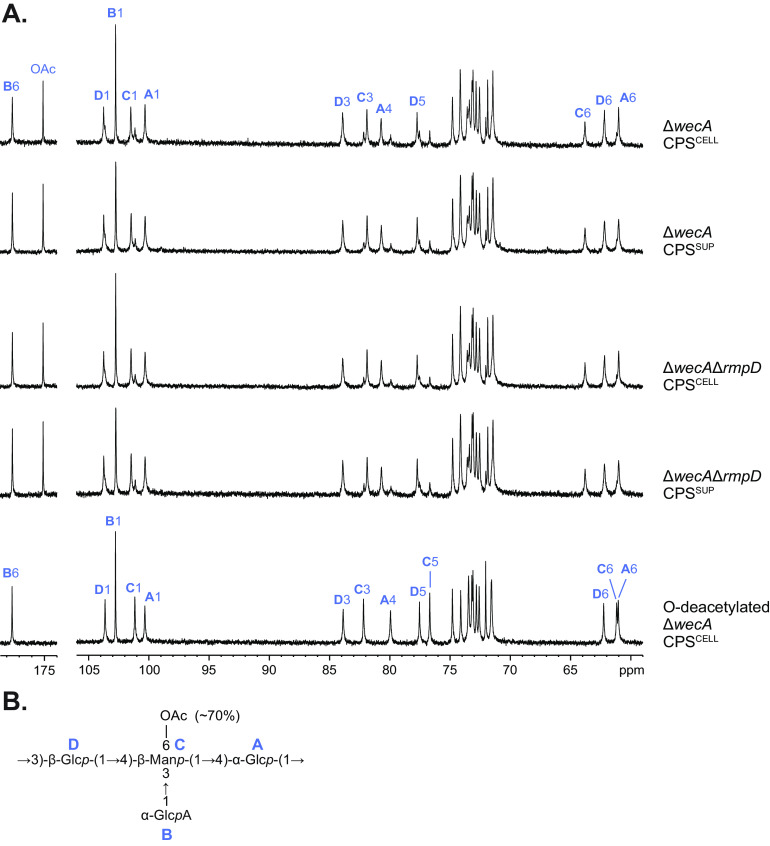
Parts of the ^13^C NMR spectra of CPS^CELL^ and CPS^SUP^ samples from K. pneumoniae with and without *rmpD.* (A) CPS samples were prepared from KPPR1S Δ*wecA* and Δ*wecA*Δ*rmpD* and analyzed after sonication and gel filtration chromatography. The *O*-deacetylated CPS^CELL^ from Δ*wecA* was obtained by treatment with aqueous ammonia. The ^13^C NMR spectra of native CPS samples are essentially identical and demonstrate structural heterogeneity due to nonstoichiometric *O*-acetylation of the Man residue. The changed ^13^C NMR spectrum of *O*-deacetylated CPS^CELL^ corresponded to a homogeneous CPS with the published K2 structure. (B) Structure of the CPS from KPPR1S. Blue letters refer to the peaks associated with the four sugars in the CPS repeat unit structure.

Detailed NMR analysis was performed on sonicated CPS^CELL^ samples from the Δ*wecA* mutant. The results revealed a repeat-unit structure corresponding to published reports for the K2 CPS (40), with one exception; the KPPR1S CPS contained an *O*-acetyl group absent in the published structure. Consistent with this observation, the KPPR1S *cps* locus contains a gene designated *orf13*, which encodes a putative acetyltransferase. In K. pneumoniae strain 52.145, the strain from which the K2 capsule structure was determined originally (40), the corresponding gene possesses a frameshift mutation and is predicted to encode a truncated (presumably nonfunctional) protein (see Fig. S2 in the supplemental material). The ^13^C NMR spectrum of CPS (Fig. 2) demonstrated structural heterogeneity, which was caused by nonstoichiometric *O*-acetylation (the signal for *O*-acetyl group CH_3_ is at δ_H_/δ_C_ 2.18/21.6). Two-dimensional (2D) correlation spectroscopy (COSY) and total correlation spectroscopy (TOCSY) spectra revealed spin systems for three sugars having *gluco*-configuration (Glc **A**, GlcA **B**, and Glc **D**) and one sugar having *manno*-configuration (Man **C**). ^13^C NMR chemical shifts were assigned based on ^1^H,^13^C heteronuclear single quantum coherence (HSQC), HSQC-TOCSY, and heteronuclear multiple bond correlation (HMBC) experiments (Table 1). The α-configuration of residue **A** was determined based on the position of **A** C-5 at δ 71.5. As judged by the *J*_1,2_ coupling constant of 3.6 Hz, GlcA **B** is α-linked, whereas Glc **D** is β-linked (*J*_1,2_ 7.7 Hz).

**TABLE 1:** ^1^H and ^13^C NMR chemical shifts (δ, ppm) of sonicated, AcOH-treated CPS^CELL^ isolated from *K. pneumoniae ΔwecA* in native and O-deacetylated forms

Sugar residue		*H-1* C-1	*H-2* C-2	*H-3* C-3	*H-4* C-4	*H-5* C-5	*H-6a* C-6	*H-6b*
*native CPS*								
→4)-α-d-Glc*p*-(1→	**A**	*5.29*	*3.60*	*3.87*	*3.67*	*4.06*	*3.77*	*3.77*
		100.3	72.5	72.8	80.7	71.5	61.0	
α-d-Glc*p*A-(1→	**B**	*5.17*	*3.54*	*3.79*	*3.51*	*4.10*		
		102.8	73.1	74.8	73.2	74.1	177.6	
→3,4)-β-d-Man*p*6OAc-(1^*a*^→	**C**	*4.79*	*4.24*	*3.87*	*4.10*	*3.80*	*4.29*	*4.70*
		101.5	71.9	81.9	73.6	74.2	63.8	
→3)-β-d-Glc*p*-(1→	**D**	*4.48*	*3.39*	*3.62*	*3.53*	*3.52*	*3.75*	*3.97*
		103.8	73.4	83.9	71.5	77.7	62.2	
*O-deacetylated CPS*								
→4)-α-d-Glc*p*-(1→	**A**	*5.30*	*3.61*	*3.89*	*3.70*	*4.07*	*3.77*	*3.77*
		100.3	72.6	72.8	79.9	71.6	61.0	
α-d-Glc*p*A-(1→	**B**	*5.16*	*3.54*	*3.79*	*3.50*	*4.10*		
		102.8	73.1	74.8	73.2	74.1	177.6	
→3,4)-β-d-Man*p*-(1→	**C**	*4.78*	*4.23*	*3.85*	*4.06*	*3.59*	*3.84*	*4.02*
		101.2	72.0	82.2	73.5	76.7	61.2	
→3)-β-d-Glc*p*-(1→	**D**	*4.53*	*3.39*	*3.64*	*3.53*	*3.54*	*3.74*	*3.98*
		103.6	73.4	83.9	71.5	77.5	62.2	

a The chemical shifts for the O-acetyl group are δ_H_ 2.18; δ_C_ 21.6 (CH_3_) and 175.1 (CO).

10.1128/mbio.00800-23.2 FIG S2 Nucleotide and amino acid alignment of predicted Orf13 from KPPR1S and Kp52.145. *orf13* of the capsule locus encodes a putative acyltransferase that is likely the source of the acetylated mannose moiety. This moiety was absent in the original description of the K2 capsule in Kp52.145, probably due to a frameshift in *orf13* (2-nucleotide [nt] deletion; red box) which introduced a premature stop codon in this strain. Download FIG S2, file, 1 MB. Copyright © 2023 Ovchinnikova et al. 2023 Ovchinnikova et al. https://creativecommons.org/licenses/by/4.0/ This content is distributed under the terms of the Creative Commons Attribution 4.0 International license.

The relatively low-field positions of the signals for **A** C-4, **C** C-3, **C** C-4, and **D** C-3, compared with their positions in nonsubstituted monosaccharides, demonstrated the modes of substitution of these residues. The signals for **B** C-2 to C-5 are characteristic of nonsubstituted α-Glc*p*A. The sequence of the sugar residues in the repeat unit was determined by an HMBC experiment, which showed the following interresidue correlation between anomeric protons and linkage carbons: **A** H-1/**D** C-3, **D** H-1/**C** C-4, **C** H-1/**A** C-4, and **B** H-1/**C** C-3. The position of the *O*-acetyl group at C-6 of Man **C** was confirmed by HMBC correlations of the carboxyl carbon and methyl protons of the *O*-acetyl group with **C** H-6a, **C** H-6b, and **C** C-6 at δ 175.1/4.29, 175.1/4.70, and 2.18/63.8, respectively. To confirm the structure and to assist with the assignment of a minor series of signals originating from non-*O*-acetylated repeat units, the CPS sample was chemically *O*-deacetylated and studied by 1D and 2D NMR as described above. The chemical shifts of *O*-deacetylated CPS (Table 1) were in agreement with the published data for K2 CPS (40). A comparison of the HSQC spectra from native and *O*-deacetylated CPS samples showed a significant shift of the signals for **C** C-6/H-6a, **C** C-6/H-6b from δ 63.8/4.29, 63.8/4.70 to δ 61.2/3.84, 61.2/4.02, which was attributed to the deshielding effect of the *O*-acetyl group. Accordingly, the signal for **C** C-5 shifted downfield from δ 74.2 to 76.7. The chemical shift of δ 76.7 for C-5 indicated the β-configuration of Man residues. As judged by the integral intensities of protons **C** H-1 and **C** H-6b in the ^1^H NMR spectrum of native CPS (Fig. 1), ~70% of the Man residues are *O*-acetylated. Thus, the Δ*wecA* strain produces CPS that differs from the published K2 CPS structure only by nonstoichiometrical *O*-acetylation of Man residues at position 6; the two subtle variants would be expected to be serologically cross-reactive.

Analyses of the ^1^H NMR (Fig. 1), ^13^C NMR (Fig. 2), and HSQC spectra of CPS^CELL^ and CPS^SUP^ from both the Δ*wecA* and Δ*wecA*Δ*rmpD* strains revealed that these polysaccharides possessed the same repeat-unit structure and the same (~70%) degree of *O*-acetylation. From these results, K2 CPS is the sole polysaccharide in the cell-bound and cell-free polymer samples derived from the HMV^Pos^ and HMV^Neg^ strains of KPPR1S (Fig. 2).

### RmpD alters the CPS profile in K. pneumoniae and in E. coli.

**FIG 3:**
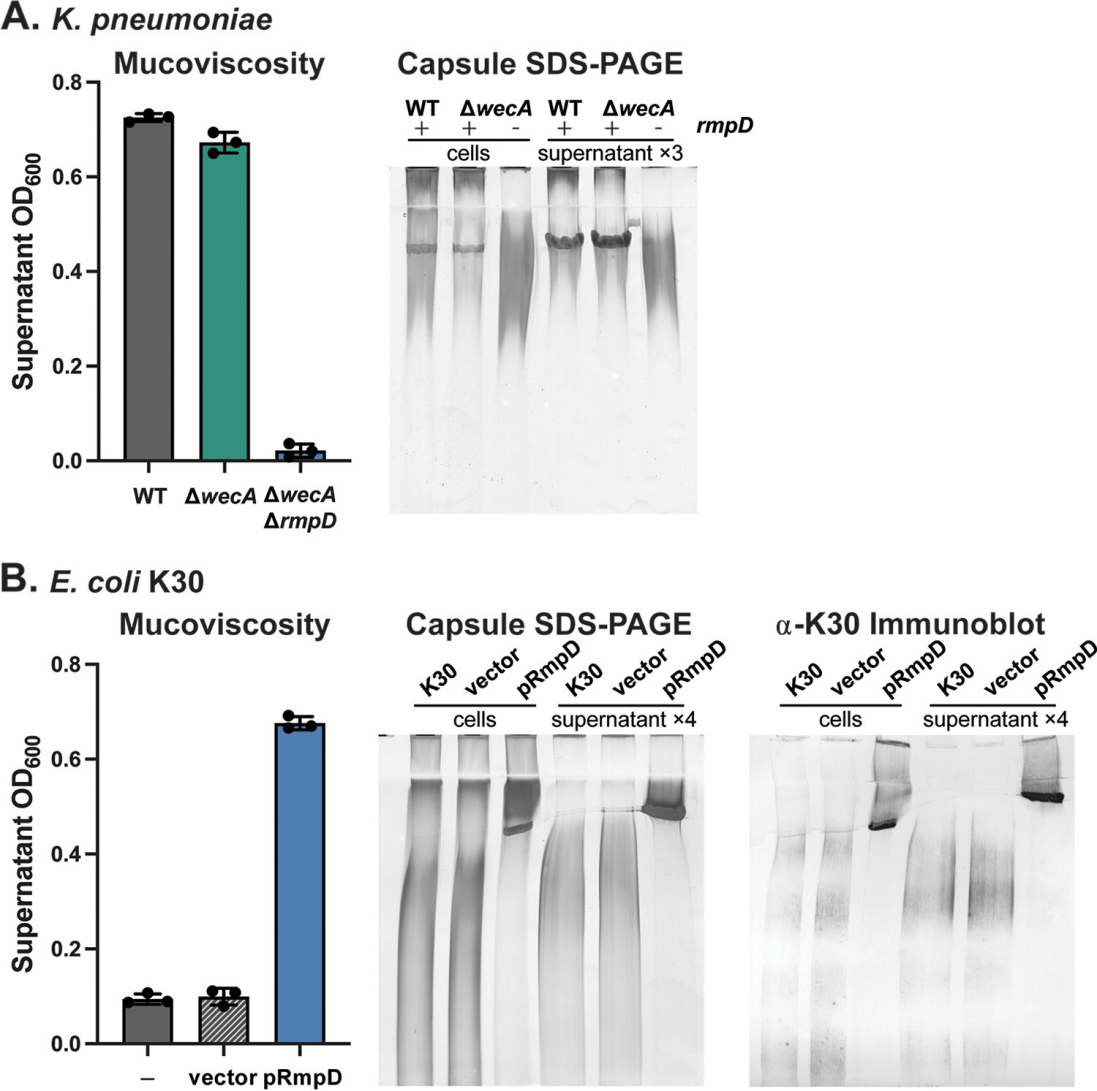
Mucoviscosity and CPS profiles of K. pneumoniae and E. coli K30 expressing *rmpD*. Saturated overnight cultures of K. pneumoniae (A) and E. coli K30 (B) were subcultured into fresh M9-CAA medium and were grown for 6 h at 37°C. Mucoviscosity was evaluated by the sedimentation assay as described in the Materials and Methods. Data are represented as the average ± standard deviation of three biological replicates. Proteinase K-treated cell lysates and culture supernatant samples were separated by SDS-PAGE, and stained first with alcian blue and then with silver as described in the Materials and Methods. Note that the amounts loaded for the supernatant samples are 3 or 4 times higher than those for the corresponding cell samples. E. coli lysates and supernatants were separated by SDS-PAGE, transferred to nylon, and probed with α-K30 antibodies. The E. coli K30 strain was transformed with pWQ743 (vector) or pWQ1126 (pRmpD). Expression of *rmpD* was induced by addition of 100 ng/mL ATc at the time of subculture.

The results above establish that the HMV phenotype is not due to the production of a new polysaccharide nor to the modification of the existing CPS chemical structure. The solution properties of microbial biopolymers are dictated by chemical structure, molecular weight, and intrinsic viscosity parameters (41). Having ruled out chemical differences as an explanation for the HMV phenotype, our attention turned to CPS chain length. Since the viscosity of the K2 CPS is increased when *rmpD* is expressed (33), the effect on chain length was assessed by SDS-PAGE using samples from cultures simultaneously tested for the HMV phenotype with the sedimentation assay (Fig. 3A). This analysis showed that CPS^CELL^ samples from wild-type K. pneumoniae KPPR1S and the Δ*wecA* mutant both present as a “smear” of stained molecules with heterogeneous sizes, together with a distinct band representing a “modal” cluster of molecules of similar size. The modal cluster was enhanced in the CPS^SUP^ fraction. In contrast, the modal cluster was absent from the CPS^CELL^ and CPS^SUP^ samples from the Δ*wecA*Δ*rmpD* mutant. These data indicate that RmpD has a major impact on the distribution of CPS chain lengths and is specifically correlated with the production of a modal cluster of molecules.

Previous studies reported that the HMV phenotype is observed in K. pneumoniae isolates with different K antigens and is therefore independent of CPS structure (32). Therefore, to further investigate the role of RmpD, *rmpD* was expressed in E. coli serotype K30, the prototype for biochemical and physiological studies of group 1 capsule production (26). While E. coli K30 and K. pneumoniae serotypes share the same assembly and export machinery, using key conserved gene products, they differ in the regulation of *cps* expression. E. coli group 1 capsule producers lack a direct transcriptional regulation of their *cps* operons by the Rcs regulon (42), and an analysis of genome sequences indicated no *rmp* genes are carried in the K30 genome. This information provided an opportunity to assess RmpD activity in a naive background.

E. coli K30 colonies are visibly less mucoid than HMV^Pos^ K. pneumoniae, but the transformation of a plasmid carrying *rmpD* increased the mucoid appearance of the colonies and resulted in an HMV^Pos^ phenotype detected in the sedimentation assay (Fig. 3B). This phenotype correlated with a substantial change in the SDS-PAGE profile of the CPS (Fig. 3B). While the wild-type strain and vector control showed a heterogenous range of CPS chain lengths, the *rmpD* transformant produced CPS with a substantially higher overall average size, as well as a homogeneous modal cluster not observed previously in E. coli K30 strains and mutants under any condition. E. coli K30 has the genetic capacity to produce a single K antigen of a known structure (43), and although the chemical structure of the product was not determined here, the modal cluster reacted with validated anti-K30 antibodies, confirming the identity of this material as an authentic K30 antigen (Fig. 3B). These results indicate that RmpD can confer the HMV phenotype by regulating the chain-length distribution of CPS in different species, independent of other capsule regulatory factors that might exist in K. pneumoniae. The data also imply that RmpD operates via one of the conserved components in the capsule assembly export pathway.

### RmpD interacts with Wzc.

RmpD is a small protein of about 49 to 58 amino acids (aa), depending on the strain (58 aa in KPPR1S), that is predicted to be comprised of an N-terminal transmembrane helix and a C-terminal domain possessing several conserved positively charged residues (33). An analysis of RmpD fused to either β-galactosidase (β-gal) or alkaline phosphatase (AP) suggested that the C terminus of RmpD is located in the cytoplasm; a strain producing RmpD-β-gal showed β-gal activity, but a strain producing RmpD-AP did not show AP activity (see Fig. S3 in the supplemental material). Although there is some diversity in the sequences of *rmpD* genes from different K. pneumoniae isolates, the overall architectures of the predicted transmembrane domain and charged C-terminal domain are conserved, and multiple *rmpD* alleles were equally capable of conferring the HMV phenotype to a KPPR1S *rmpD* mutant (33). As a small protein, RmpD is likely to exert its effect by influencing the activity of another protein (44). Therefore, identifying the interacting partner should shed light on how RmpD results in the HMV phenotype.

10.1128/mbio.00800-23.3 FIG S3 Topology analysis of RmpD. Genes encoding translational fusions of *rmpD* to *phoA* (top) or *lacZ* (bottom) were cloned into pMWO-078 and expressed in KPPR1S. Alkaline phosphatase and β-galactosidase assays were performed as described previously (84) using p-Nitrophenyl Phosphate (PNPP) or ortho-Nitrophenyl-β-galactoside (ONPG) as the substrates, respectively. Download FIG S3, file, 0.4 MB. Copyright © 2023 Ovchinnikova et al. 2023 Ovchinnikova et al. https://creativecommons.org/licenses/by/4.0/ This content is distributed under the terms of the Creative Commons Attribution 4.0 International license.

As the capsule is required for HMV and RmpD appears to be an inner membrane protein, we first considered membrane proteins involved in capsule biosynthesis and export as potential interacting partners for RmpD (see Fig. 7B for a model of the synthesis and export components). WcaJ is the initiating phosphoglycosyltransferase that catalyzes the first step in capsule biosynthesis in K. pneumoniae (45). Wzx and Wzy are the flippase and polymerase, respectively, which define the Wzy-dependent mechanism; Wzx moves the undecaprenyl diphosphate-linked CPS repeat units from the cytoplasm to the periplasm, and Wzy polymerizes the repeat units (27). The genes encoding enzymes for repeat unit synthesis vary with the CPS product structures, and *wzy* and *wzx* genes have serotype-specific sequences that encode proteins with conserved predicted secondary structures and functions. In all K. pneumoniae K serotypes (and in E. coli isolates with group 1 CPS), the variable biosynthesis gene block is preceded by a block of four highly conserved genes, as follows: *wzi-wza-wzb-wzc*. The roles of the corresponding proteins are known to various extents (reviewed in references 26 and 27). Wzi is an outer membrane β-barrel protein that is implicated in the surface organization of the capsule layer. Wza is the outer membrane translocon for CPS export and extends into the periplasm where it is thought to interact with Wzc. Wzc is an inner membrane tyrosine kinase protein involved in CPS export, and its presence is required to sustain Wzy-dependent polymerization. Wzc is dephosphorylated by Wzb, and the cycling of autophosphorylation/dephosphorylation is critical for CPS polymerization and export through the Wzc-Wza complex.

**FIG 4:**
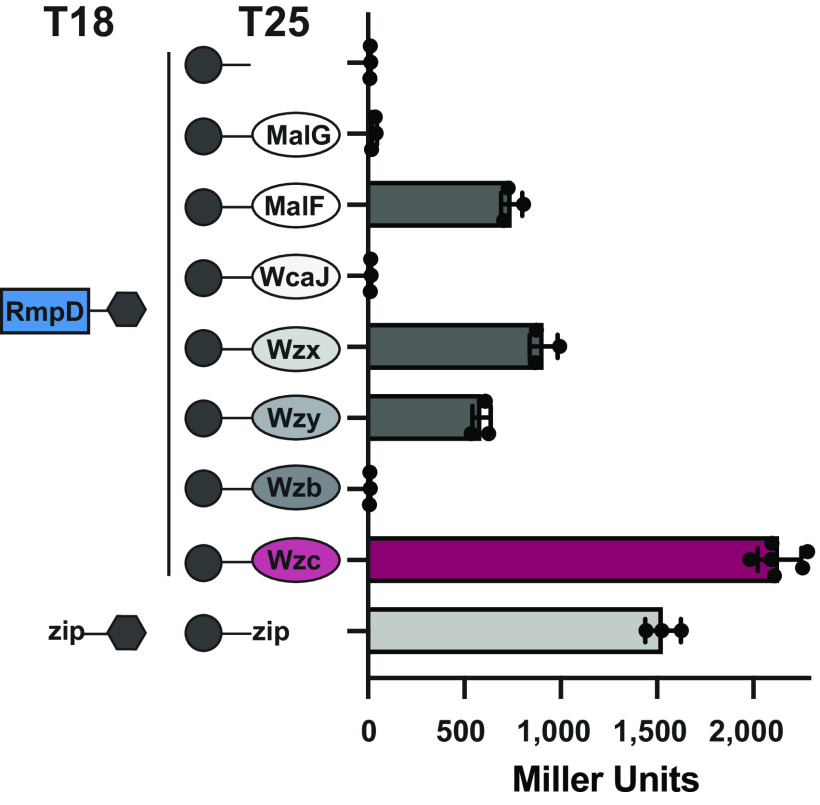
Bacterial two-hybrid analysis of RmpD with capsule biosynthesis and export proteins. Plasmids expressing *rmpD-T18* (pLPT015) and *T25* fusions to the N terminus of the indicated CPS component (pLPT013, 020, 010, 057, and 047) were transformed into BTH101, an E. coli *cya* mutant. β-Galactosidase assays were performed as described in the Materials and Methods. The positive-control plasmids contain leucine zipper (zip) domains and averaged 1,532 Miller units (MU). The vector control is pKT25 (average of 1 MU), and the membrane-bound MalF and MalG are controls for nonspecific interactions with membrane proteins. Additional controls (vector-construct pairs, RmpD-T18 with C-terminally tagged CPS proteins) are provided in Fig. S4.

An initial assessment of the ability of capsule biosynthesis components to interact with RmpD was done using the bacterial two hybrid (BACTH) system (46, 47). Capsule synthesis proteins in or at the cytoplasmic membrane were targeted due to the localization of RmpD. Because inner membrane proteins can exhibit nonspecific interactions due to proximity, we included control experiments with MalG-T25 and MalF-T25 (48, 49). Results from this assay reported no convincing specific interaction between RmpD and WcaJ, Wzx, Wzy, or Wzb. Although it is difficult to interpret negative results with two-hybrid strategies due to potential misfolding or steric hindrance caused by the fused reporters, compelling data for an interaction was observed between RmpD and Wzc. β-Gal activity from strains with RmpD and Wzc was ~2,150 Miller units (MU), which is well above that from the positive-control (zip-zip) plasmids (~1,500 MU) (Fig. 4). β-Gal activity from strains with RmpD and Wzy or Wzx was similar to that observed with MalF at less than 50% of the zip-zip plasmids (ranged 600 to 900 MU). These results may indicate indirect interactions due to proximity in the membrane, but we cannot rule out potential direct interactions with Wzy or Wzx. Strains with RmpD and WcaJ or Wzb and all other control samples (see Fig. S4 in the supplemental material) gave values lower than 10% of the zip-zip controls.

10.1128/mbio.00800-23.4 FIG S4 Additional control samples for the bacterial two-hybrid assay. The assay was performed as described in Fig. 4. Top, T18-RmpD (pLPT023) or pUT18 were paired with Wzc-T25 (pLPT014) or pKT25. Bottom, RmpD-T18 (pLPT015) was paired with C-terminally fused constructs in pKNT25 (pLPT014, 019, 012, 062, and 048). Positive-control zip-zip plasmids averaged 1,740 Miller units (set to 100), and all other samples were normalized to this average. Download FIG S4, file, 0.4 MB. Copyright © 2023 Ovchinnikova et al. 2023 Ovchinnikova et al. https://creativecommons.org/licenses/by/4.0/ This content is distributed under the terms of the Creative Commons Attribution 4.0 International license.

**FIG 5:**
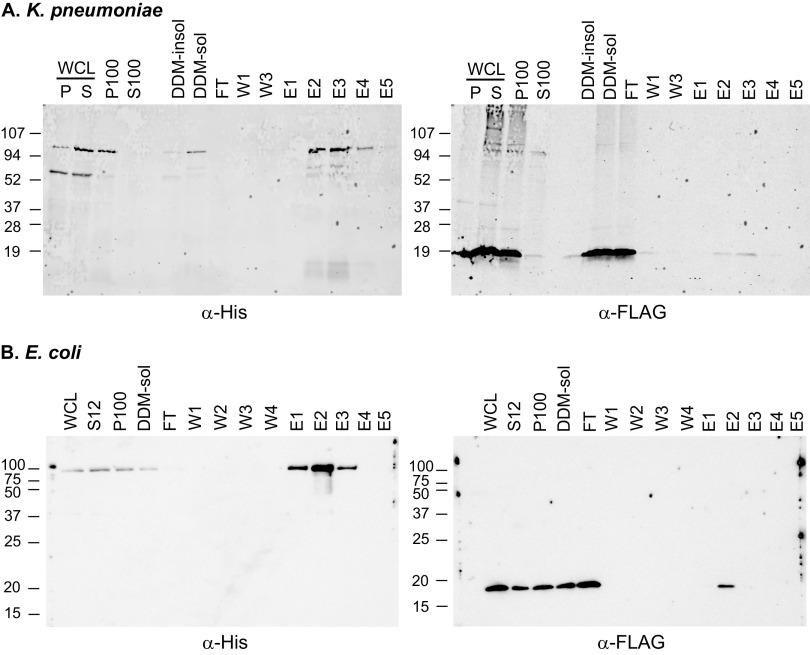
Copurification of RmpD with Wzc from K. pneumoniae (A) or E. coli K30 (B). E. coli BL21(DE3) was cotransformed with pKW197 (pRmpD-FLAG_2_) and pLPT052 (pHis_6_-Wzc^K541M^) for K. pneumoniae or with pKW197 and pBN008 (pWzc^K540M^-His_6_) for E. coli K30. Cultures were grown, induced, purified, and probed as described in the Materials and Methods. In A, a single gel was run, transferred, and simultaneously probed with mouse α-His and rabbit α-FLAG antibodies, followed by simultaneous incubation with goat α-mouse and goat α-rabbit fluorescently labeled secondary antibodies. Single-channel images are presented; the original dual-color image is in Fig. S5. In B, two identical gels were run and transferred, with one probed with mouse α-His and the other with mouse α-FLAG and then with HRP-conjugated goat α-mouse secondary antibody. WCL, whole-cell lysate (separated centrifugation at 12,000 × *g* spin in A; P, pellet; S, supernatant); P100 and S100, pellet and supernatant following centrifugation at 100,000 × *g*; DDM-insol and DDM-sol, pellet and supernatant following incubation with 1% DDM and centrifugation at 100,000 × *g*; W1 to 4, washes; E1 to 5, elution fractions; S12, supernatant following centrifugation at 12,000 × *g*. Numbers to the left of each blot indicate molecular weight markers in kDa. RmpD-FLAG_2_ is predicted to be 9.3 kDa, and His_6_-tagged Wzc is calculated to be about 82 kDa. Both proteins migrate slower than predicted based on size, which may be due to unknown physical properties. The additional band migrating above the 52-kDa band is likely a nonspecific protein as it is also present in control blots lacking Wzc (Fig. S5A).

To provide further support for an interaction between RmpD and Wzc, a protein copurification approach was used. Plasmids encoding RmpD-FLAG_2_ and His_6_-Wzc were cotransformed into E. coli BL21, and His_6_-tagged proteins were isolated from detergent extracts by chromatography on Ni-nitrilotriacetic acid (NTA) resin. The presence of Wzc and RmpD in the purified elution fractions was determined by Western blot analysis using an α-His antibody to detect Wzc and an α-FLAG antibody to detect RmpD. Wild-type *wzc* (Wzc^WT^) as well as a *wzc* mutant encoding a K541M substitution (Wzc^K541M^) were tested. The Wzc^K541M^ mutation in Wzc from KPPR1S is equivalent to the K540M mutation in the E. coli Wzc that lacks tyrosine kinase activity and stabilizes the octameric state, whereas Wzc^WT^ cycles between octamer and monomer forms (50). In samples with His_6_-Wzc^K541M^ and RmpD-FLAG_2_, both proteins were detected in the elution fractions (Fig. 5A). No RmpD-FLAG_2_ was detected from lysates lacking His_6_-Wzc (see Fig. S5A in the supplemental material), indicating that the RmpD interaction was specific. In contrast to the Wzc^K541M^ mutant, samples with His_6_-Wzc^WT^ and RmpD-FLAG_2_ showed no reliable detection of RmpD in the elution fractions (Fig. S5C). These data suggest RmpD preferentially interacts with the nonphosphorylated form of Wzc, which favors stable octamers.

10.1128/mbio.00800-23.5 FIG S5 Copurification assays for RmpD with pET28a and Wzc^WT^. E. coli strain BL21 was cotransformed with pKW197 (pRmpD-FLAG_2_) and pET28a (A), pKW197 only (B), pLPT045 (K. pneumoniae pHis_6_-Wzc^WT^) (C), or pBRC901 (E. coli K30 pWzc^WT^-His_6_) (D). Preparation of the samples is as described in the Materials and Methods and in Fig. 5. Note the very faint band in elutions 2 and 3 with the E. coli K30 Wzc. Also note the absence of any detectable RmpD-FLAG_2_ in the elution lanes in A with pET28a (no His_6_-Wzc) and in B with pKW197 alone. The blot in E is the same as that in Fig. 5A (pRmpD-FLAG_2_ + pHis_6_-Wzc^K541M^), presented as the dual-color image for comparison with control blots. The band migrating >52 kDa is likely a nonspecific band as there is no His-tagged Wzc in these samples. Download FIG S5, file, 5.9 MB. Copyright © 2023 Ovchinnikova et al. 2023 Ovchinnikova et al. https://creativecommons.org/licenses/by/4.0/ This content is distributed under the terms of the Creative Commons Attribution 4.0 International license.

Given that RmpD conferred HMV and influenced CPS chain length in E. coli K30, we tested if RmpD could also be copurified with the E. coli Wzc-His_6_ using the same strategy. We observed that RmpD-FLAG_2_ was present in the elution fractions from samples containing Wzc^K540M^ (Fig. 5B). As with K. pneumoniae Wzc, RmpD was not reliably detected from samples containing E. coli Wzc^WT^ (Fig. S5D), nor was RmpD detected in the control strain lacking E. coli Wzc (Fig. S5B).

### Effect of *wzc* point mutations on mucoviscosity.

**FIG 6:**
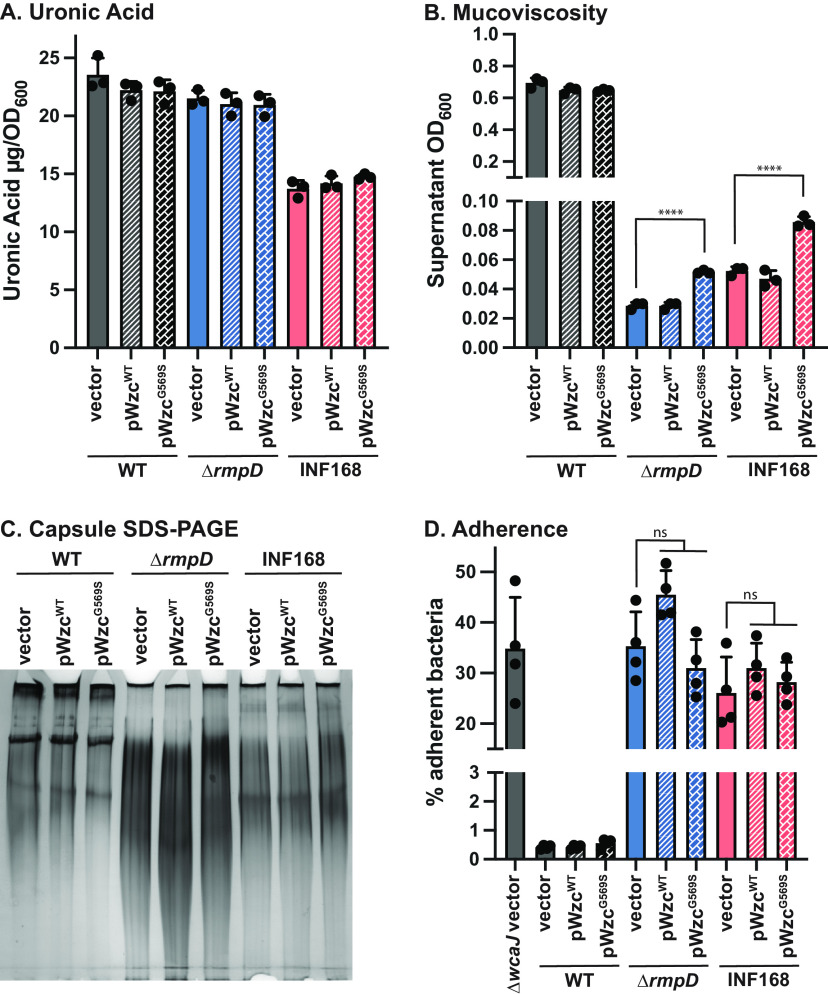
Mutation in Wzc increases mucoviscosity and capsule chain length. KPPR1S wild type (WT), Δ*rmpD*, or INF168 transformed with the indicated plasmids were grown for 5.5 h with 100 ng/mL ATc to induce *wzc* expression. (A) Uronic acid amounts, normalized to culture OD_600_. There were no significant differences between any strain with indicated plasmids (one-way ANOVA with Tukey’s posttest). (B) Mucoviscosity was measured with the sedimentation assay. One-way ANOVA with Tukey’s posttest was used to assess significance. ****, *P* < 0.0001 compared with the vector control. (C) Proteinase K-treated cell lysates from the same cultures in A were separated by SDS-PAGE, and stained with alcian blue and then with silver. The samples from Δ*rmpD* and INF168 have approximately twice the amount of material loaded compared with those of the WT. The gel image was captured with a Syngene G-Box XT4 with autodetection settings. (D) Adherence to J774A.1 cells was determined as in the Materials and Methods. There were no significant differences comparing vector controls to strains with *wzc* plasmids (one-way ANOVA with Tukey’s posttest). ns, not significant.

Recently Ernst et al. (51) reported that some cKp K. pneumoniae bloodstream isolates belonging to sequence type 258 (ST258) exhibited a mucoid colony phenotype on blood agar plates. These ST258 isolates did not contain *rmpADC*. Instead, the mucoid colony phenotype, as well as an increase in CPS, was associated with a G565S point mutation in Wzc. Residue 565 is located near the predicted Walker A’ box and is adjacent to a tyrosine residue targeted for autophosphorylation. To determine if this point mutation could alter the mucoidy phenotype in a similar manner to RmpD, we transformed strains with plasmids expressing wild-type *wzc* (pWzc^WT^) or *wzc* with the G569S mutation (pWzc^G569S^); G569S is the equivalent mutation in KPPR1S to that identified in reference 51. These strains were then assayed for HMV, CPS production, and CPS chain length. UA and HMV were restored in the Δ*wzc* strain by pWzc^G569S^ to the same levels as pWzc^WT^, indicating the mutant protein is functional (Fig. S6). The amount of CPS produced (based on uronic acid [UA] content) was the same among the wild-type and Δ*rmpD* strains expressing the *wzc* variants (Fig. 6A); this finding is consistent with data showing that the loss of *rmpD* does not impact the amount of CPS produced (33). Wild-type KPPR1S transformed with pWzc^WT^, pWzc^G569S^, or vector had similar sedimentation properties (supernatant [Sup] optical density at 600 nm [OD_600_] of ~0.6) (Fig. 6B). However, when tested in the Δ*rmpD* mutant, pWzc^G569S^ increased the Sup OD_600_ by 2- to 3-fold (~0.06) compared with Δ*rmpD* with either pWzc^WT^ or the vector (Sup OD_600_ of ~0.02). Although only a slight difference in Sup OD_600_, this difference was consistently observed and was statistically significant. The SDS-PAGE analysis of CPS from Δ*rmpD* with pWzc^G569S^ revealed a shift in the CPS chain length profile (Fig. 6C). This profile showed a heterogeneous distribution like Δ*rmpD* with vector. However, the overall pattern observed from Δr*mpD* with pWzc^G569S^ indicated a subtle increase in the average chain length compared to with that of Δ*rmpD* with pWzc^WT^ or the vector (note lanes with 2× volume loaded), but the introduction of pWzc^G569S^ did not restore the modal cluster characteristic of RmpD activity. To ascertain if strain background influenced the impact of the G569S mutation, we transformed a classical isolate (INF168) with pWzc^WT^ and pWzc^G569S^. With pWzc^G569S^, we observed a similarly small increase in Sup OD_600_ (Fig. 6B) but no appreciable change in CPS chain length (Fig. 6C). It is important to note that these strains still have wild-type *wzc* that could temper the effect of Wzc^G569S^ compared with what was reported by Ernst et al. (51). Thus, while the increased mucoidy observed in clinical isolates with *wzc* mutations may be due to the production of longer CPS chains, it is distinct from the modal cluster observed from strains with *rmpD*.

10.1128/mbio.00800-23.6 FIG S6 KPPR1S wild type or Δ*wzc* transformed with the indicated plasmids were grown for 5.5 h with 100 ng/mL ATc to induce *wzc* expression and assayed for UA (A) and mucoviscosity (B) as in Fig. 6. Download FIG S6, file, 0.4 MB. Copyright © 2023 Ovchinnikova et al. 2023 Ovchinnikova et al. https://creativecommons.org/licenses/by/4.0/ This content is distributed under the terms of the Creative Commons Attribution 4.0 International license.

CPS is known to inhibit adherence to and phagocytosis by host cells, and the avoidance of adherence is considered a virulence attribute, as interactions with host cells are an important step toward phagocytosis and clearance of K. pneumoniae (1). Experiments with the mouse macrophage-like J774A.1 cells showed that the Δ*rmpD* mutant displayed similar adherence as mutants lacking CPS, with both showing a >15-fold increase in adherent bacteria compared with that of the wild-type (33). This result suggested that HMV also plays a critical role in blocking adherence to host cells. To determine if the slight increase in sedimentation resistance and increased chain length observed in the Δ*rmpD* strain with pWzc^G569S^ impacted adherence, we performed an adherence assay with J774A.1 cells. The wild-type strain showed very low adherence, at <1%, while the capsule-deficient strain (Δ*wcaJ*) showed about 35% adherence. The Δ*rmpD* mutant and the classical strain showed similar adherence with pWzc^WT^ or pWzc^G569S^ as with the vector control at ~35% for Δ*rmpD* and 25% for INF168 (Fig. 6D). These data suggest that a more robust change in capsule chain length or modality, like that observed with RmpD, is necessary to enhance this virulence attribute.

## DISCUSSION

More than 30 years ago, highly mucoid strains of K. pneumoniae were reported and the mucoid colony morphology was linked to the presence of a large plasmid encoding RmpA (17). This report and subsequent reports demonstrate that the mucoid colony phenotype is associated with an increase in the virulence of K. pneumoniae (17, 20, 31, 33, 34, 52). The mucoid colony and HMV phenotypes have been linked to the capsule (17, 33, 34), but it was not known if the polysaccharide(s) conferring this phenotype was a modified form of the capsule or possibly a distinct polysaccharide that required some aspect of capsule biosynthesis. Recently, we identified a small protein, RmpD, that is required for the HMV phenotype but that does not impact the amount of CPS produced. Here, our analysis of K. pneumoniae with and without *rmpD* demonstrated that the structure of the polysaccharide conferring HMV is identical to the structure of the capsule (K2) produced by this strain. An unanticipated observation was that approximately 70% of the K2 CPS was *O*-acetylated at the mannose residue, which may have implications for studies investigating therapeutic and vaccine strategies that target the capsule (53). While the polysaccharide structure was identical, we found that in the presence of RmpD, the CPS chain length distribution is substantially different; the average chain length increased and a large amount of the total chains fall within a more uniform size range. The resulting modal cluster of CPS is not detected in the absence of RmpD. To date, the *rmpADC* locus has been identified only in K. pneumoniae, but examination of E. coli K30 expressing *rmpD* from a plasmid also showed an increase in the uniformity and chain length of the capsule. This result indicates that RmpD can function in a heterologous host that produces a different CPS structure but uses the same assembly mechanism as K. pneumoniae.

Wzc showed a strong interaction with RmpD in the bacterial two-hybrid assay, and this result was validated by copurification of RmpD with Wzc. That RmpD may impact HMV via interaction with Wzc is consistent with recent reports of point mutations in *wzc* that increase the mucoidy of colonies in several different bacterial species that lack *rmpD* (51, 54–56). These mutations are typically in conserved regions of *wzc* required for the tyrosine kinase activity. In two of these reports, the *wzc* point mutation correlated with an increase in CPS chain length (54, 55). However, the precise impact of the *wzc* mutations on CPS biosynthesis differs from that of RmpD because only the expression of *rmpD* leads to a characteristic modal cluster of CPS chain lengths. In the absence of RmpD, Wzc^G569S^ led to a slight increase in supernatant OD_600_ in a sedimentation assay but did not have nearly the same impact as RmpD, and this mutation did not enhance the ability to avoid adherence to J774A.1 cells despite the slightly longer chains. A recent study comparing a large collection of cKp strains (which lack *rmpD* and are HMV^Neg^) to hvKp strains (which have *rmpD* and are HMV^Pos^) indicated that results from the sedimentation assay as a measure of HMV could distinguish cKp strains from hvKp, and a sedimentation threshold was established (57). Isolates with a supernatant OD_600_ of 0.2 or greater are considered HMV^Pos^. In the KPPR1SΔ*rmpD* strain with Wzc^G569S^, the supernatant OD_600_ was still well below this threshold and would be classified as HMV^Neg^. Thus, while some point mutations in *wzc* and the acquisition of *rmpD* are both able to increase colony mucoidy and CPS chain length, the acquisition of *rmpD* confers an HMV^Pos^ phenotype and has stronger effects on phenotypes associated with HMV, such as that observed with the adherence assay.

Wzc is an inner membrane protein with tyrosine kinase activity that had been implicated in capsule chain length control and CPS export (see references 58 and 59; reviewed in reference 26). Each monomer of Wzc possesses two transmembrane helices separated by a predominantly α-helical periplasmic domain, and the cytosolic tyrosine kinase is located at the C terminus. Recently, the structure of the dephosphorylated Wzc octamer from E. coli K30 was determined using the Wzc^K540M^ mutant that inactivates the kinase activity (50). In its unphosphorylated state, the protein favors a stable octamer with the C-terminal tyrosine-rich tail of one monomer located in the active site of the adjacent monomer to form a ring structure (50, 60, 61). Increased phosphorylation of multiple C-terminal tyrosine residues destabilizes the structure so the octameric form can no longer be isolated (50). The activities of the Wzc kinase and its cognate phosphatase, Wzb, are both essential for the polymerization and transport of the CPS polysaccharide, leading to the concept of a cycling of states being necessary during active synthesis (58, 62, 63). In the destabilized state, Wzb would be able to access and dephosphorylate the phosphotyrosine residues in the C terminus now released from the active site of the adjacent monomer to facilitate recycling. Exactly how each state of Wzc participates in biosynthesis is not yet known and interpretation is complicated by the apparent involvement of Wzc in two different stages of CPS production.

Wzc is a member of the polysaccharide copolymerase (PCP) family. The prototypical member is Wzz (PCP-1), which regulates the modality of LPS *O*-antigen glycans synthesized by a Wzy-dependent pathway resembling the CPS-biosynthesis mechanism of K. pneumoniae (reviewed in reference 38). *wzz* mutants classically result in a heterogenous chain-length distribution emphasizing shorter chains and lack defined modal clusters in SDS-PAGE profiles. Wzz shares architectural similarities with Wzc, particularly in the transmembrane regions, but lacks a tyrosine kinase and seems to form a stable octamer (50, 64, 65). That Wzz modulates the polymerization reaction is supported by reported interactions between Wzz and Wzy (66–68), and a recent study indicates that a Wzy protein resides in the membrane enclosed within the lumen of a Wzz octamer (69). The stability of the Wzz oligomer is implicated in chain-length regulation. Similarities in Wzc protein structures and mutant phenotypes (58) are consistent with the proposal that Wzc and Wzy may also interact to modulate CPS production in a related way. Export of LPS molecules to the cell surface is achieved by the Lpt machinery (reviewed in reference 70), with no apparent role for Wzz. In contrast, Wzc is believed to perform an additional role in CPS production by forming part of an export channel from the site of CPS synthesis to the cell surface. Export of CPS is achieved via interactions between Wzc and the CPS outer membrane translocon octameric Wza (reviewed in reference 27). It is believed that this interaction is susceptible to the stability of the Wzc octamer (50). Therefore, the stability of Wzc octamers may impact both the biosynthesis and export stages.

It is currently unclear how RmpD exerts its effect on CPS polymerization and the HMV phenotype. The copurification data suggest that RmpD preferentially binds to the unphosphorylated form of Wzc, but how this binding influences the oligomeric state of Wzc is not yet known. Given our current understanding of Wzc structure and function, modulation of the oligomer-monomer transition is a favored hypothesis. This modulation could potentially affect rates of export to promote the production of longer chains, but the observation from the E. coli K30 system that an inability to export has a feedback effect on CPS biosynthesis (71–73) seems to argue against this hypothesis. In contrast, the characteristic generation of chain length modality argues more for an influence on interactions between Wzy and Wzc that push the polymerization process into a more processive (and less distributive) mode. These possibilities are hard to address experimentally and require a level of structural and biochemical understanding of Wzy function and Wzy:PCP protein interactions that is currently unavailable for any glycan assembly system. These questions will form the basis for future studies.

**FIG 7:**
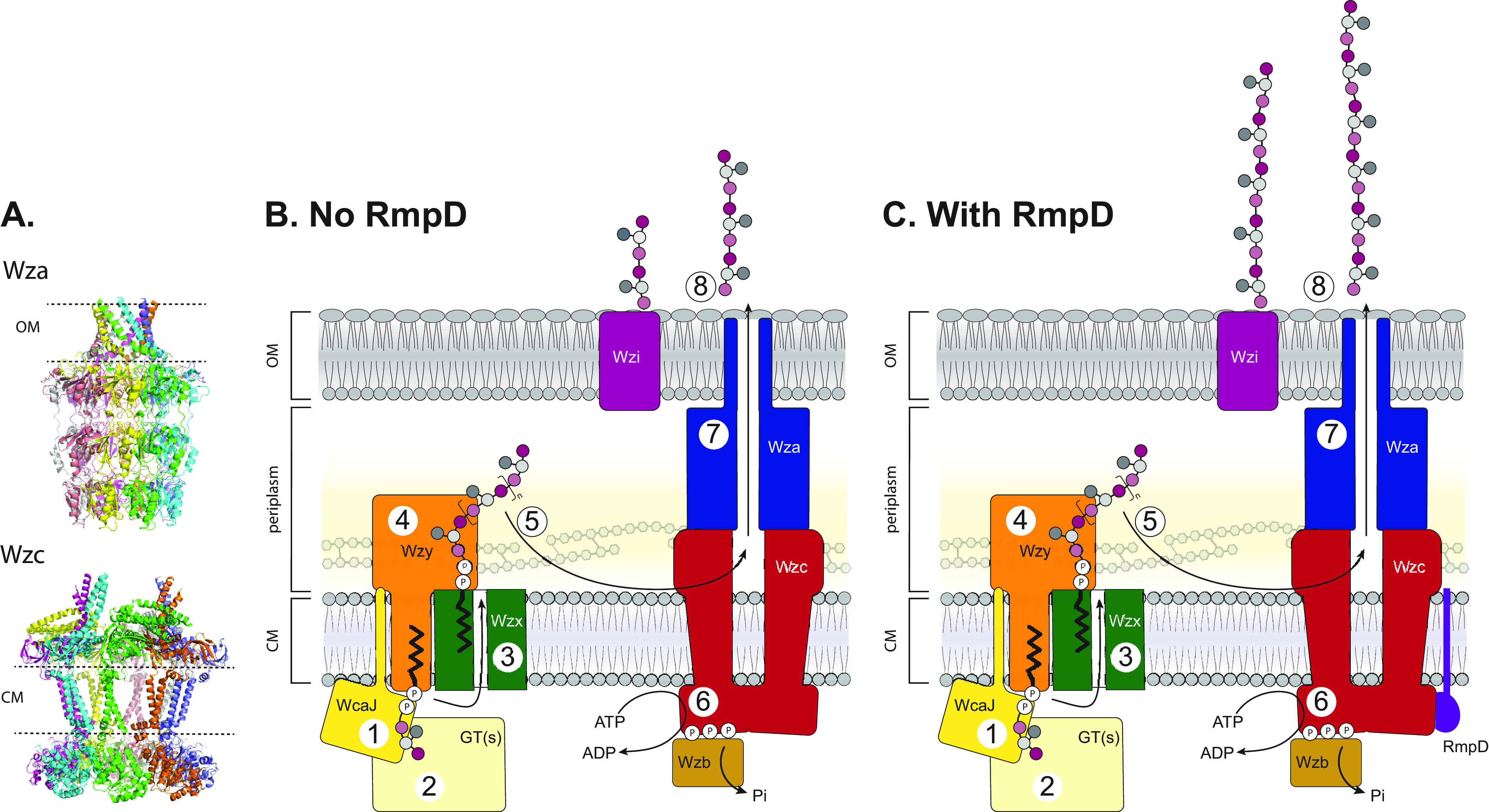
Cellular locations and known/proposed functions of the proteins required for biosynthesis and export of K. pneumoniae CPS. (A) The solved structures of Wza and Wzc multimers. (B) Synthesis of CPS repeat units is initiated on an undecaprenol phosphate acceptor by a phosphoglycosyltransferase (WcaJ, 1) and completed by the activities of serotype-specific glycosyltransferases (GTs; 2). The lipid-linked repeat units are exported via Wzx, a member of the MOPS transporter family (3) and polymerized by a Wzy homolog (4). This is followed by translocation involving a complex comprising octamers of the PCP-2a (Wzc) and OPX (Wza) proteins (5–7). Wzc plays dual roles in polymerization and translocation driven by its reversible phosphorylation (6). This likely requires direct interaction between Wzy and Wzc proteins based on data for Wzy-Wzz interactions in other systems which place Wzy within the Wzz lumen (69). Since the details and relative locations of synthesis and export-translocation proteins are not yet established by direct experiments for CPS biosynthesis, they are not inferred in the model shown here. While the capsular polymers are closely associated with the cell surface, the underlying processes for the association are not fully understood. The lectin-like activity of Wzi is involved but is likely not sufficient, and a substantial amount of the polysaccharide product is released from the cell. (C) In the presence of RmpD, CPS chains are elongated, likely through an interaction with Wzc. The location and nature of this interaction is not known. This figure is a modified version of a model published previously (26).

Collectively, the data presented here have demonstrated that a small protein, RmpD, alters Wzc-mediated CPS synthesis to produce longer polysaccharide chains of a more uniform length (Fig. 7). The production of the longer capsule chains is likely an essential factor for the HMV^Pos^ phenotype, which has important implications for pathogenesis. That RmpD can elicit an HMV^Pos^ phenotype in a heterologous host raises concerns for the spread of this phenotype to other organisms through the horizontal transfer of the large virulence plasmids that carry the *rmp* locus (23, 74), thereby potentially enhancing the virulence of other pathogens.

## MATERIALS AND METHODS

### Bacterial strains, plasmids, and growth conditions.

The strains and plasmids used in this work are listed in Table 2. Unless otherwise noted, K. pneumoniae and E. coli were cultured in LB medium (10 g tryptone, 5 g yeast extract, and 10 g NaCl per litre) at 37°C. The following antibiotics were used where appropriate at the indicated concentrations: kanamycin (Kan), 50 μg/mL; rifampin (Rif), 30 μg/mL; spectinomycin (Spt), 50 μg/mL; and carbenicillin (Amp^r^), 100 to 200 μg/mL. For the expression of genes cloned into pMWO-078 (75), 100 ng/mL anhydrous tetracycline (ATc) was added to the medium at the time of subculture unless otherwise noted. Other inducers (isopropyl-β-d-thiogalactopyranoside [IPTG] and arabinose) were added as described in the Wzc and RmpD copurification and immunoblot’ section.

**TABLE 2:** Strains and plasmids used in this work

Strain or plasmid	Relevant genotype	Reference
Strains		
E. coli		
DH5α	*F*^−^ p*80*Δ*lacZM15* Δ*(lacZYA-argF)U169 deoP recA1*, *endA1 hsdR17* (r_k_^−^m_k_^−^)	Invitrogen
S17-1λ*pir*	Tp^r^ Str^r^ *recA thi pro hsdR hsdM^+^* RP4::2-Tc::Mu::Km, Tn7 λ *pir* lysogen	87
BL21 (λDE3)	*F*^−^ *ompT hsdS_B_*(r_B_^−^m_B_^−^) *gal dcm*	NEB
E69	O9a:K30	88
K. pneumoniae		
KPPR1S	ATCC 43816, Rif^r^, Str^r^	89
VK487	KPPR1S, Δ*rmpC*	31
VK506	KPPR1S, Δ*manC*	31
VK429	KPPR1S, Δ*rmpADC*	33
VK646	KPPR1S, Δ*wcaJ*	33
VK637	KPPR1S, Δ*rmpD*	33
VK679	KPPR1S, Δ*wzc*	This work
VK685	KPPR1S, Δ*wecA*	This work
VK689	KPPR1S, Δ*rmpD ΔwecA*	This work
INF168	classical isolate, ST15, KL16	90
Plasmids		
pKAS46	Kan^r^; MobRP4 *ori*R6K, cloning vector	77
pKD4	FRT-flanked Kan^r^ cassette	78
pKD46-Spec^R^	P_araB_ λ red recombinase; *oriR101*	79
pLH29-*sacB*	Cm^r^; FLP recombinase; *sacB*	This work
pET28a	N-terminal 6×-histidine tag; T7 promoter	EMD Millipore
pMWO-078	Sp^r^; p15A *ori* expression vector, *tetO*	75
pWQ743	Amp^r^; pBR322 *ori* expression vector, *tetO*	91
pWQ1126	*rmpD* in pWQ743	This work
pLPT007/pRmpD	*rmpD* in pMWO-078	33
pLPT034	*wecA* in-frame deletion in pKAS46	This work
pUT18	Amp^r^; for C-terminal T18 fusions in BACTH	Euromedex
pUT18C	Amp^r^; for N-terminal T18 fusions in BACTH	Euromedex
pKT25	Kan^r^; for N-terminal T25 fusions in BACTH	Euromedex
pKNT25	Kan^r^; for C-terminal T25 fusions in BACTH	Euromedex
pUT18C-zip	Amp^r^; positive control for BACTH with leucine zipper domain	Euromedex
pKT25-zip	Kan^r^; positive control for BACTH with leucine zipper domain	Euromedex
pLPT013	*wzc* in pKT25	This work
pLPT020	*wzb* in pKT25	This work
pLPT010	*wzy* in pKT25	This work
pLPT057	*wzx* in pKT25	This work
pLPT047	*wcaJ* in pKT25	This work
pLPT014	*wzc* in pKNT25	This work
pLPT019	*wzb* in pKNT25	This work
pLPT012	*wzy* in pKNT25	This work
pLPT062	*wzx* in pKNT25	This work
pLPT048	*wcaJ* in pKNT25	This work
pLPT015	*rmpD* in pUT18	This work
pLPT023	*rmpD* in pUT18C	This work
pAJD1080	*malG* in pKT25; BACTH control plasmid	48
pAJD1079	*malF* in pKT25; BACTH control plasmid	48
pLPT033/pWzc^WT^	*wzc* in pMWO-078	This work
pKW204//pWzc^G569S^	wzc in pMWO-078	This work
pLPT045/pHis_6_-Wzc^WT^	KPPR1S *wzc* in pET28a	This work
pLPT052/pHis_6_-Wzc^K541M^	KPPR1S *wzc-K541M* in pET28a	This work
pBRC901/pWzc^WT^-His_6_	Amp^r^; K30 *wzc* (wild type) in pBAD24	50
pBN008/pWzc^K540M^-His_6_	Amp^r^; E69 *wzc-K540M* in pBAD24	50
pKW197	*rmpD-FLAG_2_* in pMWO-078	33
pKW201	*rmpD-lacZ* fusion in pMWO-078	This work
pKW202	*rmpD-phoA* fusion in pMWO-078	This work

### Construction of plasmids and K. pneumoniae mutants.

The primers used for plasmid and mutant construction are listed in Table S1 in the supplemental material. Vector DNA was linearized with restriction enzymes from New England BioLabs (NEB; as specified in primer sequences). Inserts for cloning were amplified from KPPR1S genomic DNA, and plasmids were constructed by Gibson assembly following the manufacturer’s protocol (NEB). For pKW201 and pKW202, the *rmpD* sequence was synthesized as a gBlock by IDT DNA (Coralville, IA), *lacZ* was amplified from pFU61, and *phoA* was amplified from pFU84 (76). pKAS46-based plasmids were transformed into E. coli S17-1 λ*-pir*. All other plasmids were transformed into E. coli DH5α. All plasmids were confirmed by sequencing at Genewiz/Azenta Life Sciences.

10.1128/mbio.00800-23.7 TABLE S1 Primers and synthetic genes used in this work. Download Table S1, file, 0.04 MB. Copyright © 2023 Ovchinnikova et al. 2023 Ovchinnikova et al. https://creativecommons.org/licenses/by/4.0/ This content is distributed under the terms of the Creative Commons Attribution 4.0 International license.

For the expression of *rmpD* in E. coli K30, *rmpD* was amplified from pLPT007 using primers OL300-F and OL300-R and ligated into pWQ743 at the BamHI and HindIII restriction sites. The resulting plasmid pWQ1126 was confirmed by restriction digest and sequencing (performed at the Advanced Analysis Centre, University of Guelph).

### Introduction of point mutations in *wzc*.

For the construction of pWzc^G569S^ (pKW204), primers LT046/KW472 and KW473/LT047 (Table S1) were used to amplify *wzc* in two pieces that were assembled into pMWO-078 by Gibson assembly. Primers KW472 and KW473 overlap and contain nucleotides coding for S569. The mutation for K541M was introduced by megaprimer PCR. Primer LT075 contains nucleotides coding for M541and was paired with LT072 to generate the megaprimer. This product was paired with LT071 to amplify the full-length *wzc* gene. The product was cloned into pET28a to generate pLPT052.

### Deletion of *wecA*.

An in-frame deletion of *wecA* was generated by homologous recombination using pKAS46 (77) as described (31). Briefly, 500-bp fragments flanking the gene were amplified and cloned into pKAS46, generating plasmid pLPT034. pLPT034 was conjugated into KPPR1S and VK637, and the deletion of *wecA* was confirmed by PCR following counterselection to verify plasmid loss.

### Deletion of *wzc*.

An in-frame deletion of *wzc* was generated using lambda red mutagenesis (78) with modifications described by Bachman et al. (79). KPPR1S with pKD46-Spec was transformed with a PCR product containing *wzc* sequences flanking the kanamycin resistance cassette amplified from pKD4 with primers LT026 and LT027 and recovered in Super Optimal broth with Catabolite repression (SOC) medium with 50 mM arabinose at 26°C overnight. The culture was plated onto LB Kan_50_ plates and incubated overnight at 37°C to select for colonies that integrated the Kan cassette and lost pKD46. To resolve the Kan cassette, integrants were transformed with pLH29-*sacB*, incubated overnight at 26°C with 1 mM IPTG to induce the FLP recombinase, then subcultured for 4 h at 37°C, and then serial diluted and plated onto LB plates with 5% sucrose to select for colonies that had lost pLH29-*sacB*. Resulting colonies were patched onto plates with Kan or chloramphenicol to verify the excision of the Kan cassette and plasmid loss, and several colonies were subjected to colony PCR and sequencing to confirm the deletion of *wzc*.

### CPS purification.

The purification scheme developed for this study is shown in Fig. S1A. Overnight cultures of KPPR1S wild-type, Δ*wecA* (VK685), and Δ*wecA*Δ*rmpD* (VK689) grown in M9-CAA were subcultured into 2 L fresh M9-CAA at a starting OD_600_ of 0.1 and grown for 6 h. The cells were harvested by centrifugation at 13,000 × *g* for 20 min to yield a loosely formed cell pellet and supernatant. Each fraction was mixed with 1/5 volume of 1% Zwittergent 3-14 detergent in 100 mM citric acid and incubated in a water bath at 50°C for 45 min with occasional mixing (80). The cells were collected by centrifugation at 13,000 × *g* for 20 min. The Zwittergent 3-14 detergent-treated supernatant derived from the initial loose cell pellet was mixed with absolute ethanol (final concentration of 80%), and CPS was allowed to precipitate for a minimum of 4 h (or overnight) at 4°C. The pellet was collected by centrifugation at 13,000 × *g* for 20 min at 4°C and was allowed to air dry before being resuspended in 125 mL 20 mM Tris-HCl (pH 8.0) and 2 mM MgCl_2_. A total of 1 mg DNase and 1 mg RNase were added, and the mixture was incubated at 37°C for 2 h. Then, 2 mg proteinase K was added and the mixture was incubated at 55°C for 2 h; the sample was then dialyzed against distilled water using 3.5-kDa molecular weight cutoff (MWCO) Spectra/Por membrane tubing, concentrated using a rotary evaporator, cleared by centrifugation at 4,500 × *g*, 10 min, and lyophilized. The crude CPS^CELL^ yields for K. pneumoniae Δ*wecA* and Δ*wecA*Δ*rmpD* were 430 and 649 mg, respectively. The Zwittergent 3-14 detergent-containing sample derived from the initial supernatant (>2-L volume) was concentrated to ~200 mL by cross-flow filtration using a Vivaflow 200 cassette containing a 10-kDa MWCO Hydrosart membrane. Further purification steps were essentially the same as described above. The crude CPS^SUP^ yields for K. pneumoniae Δ*wecA* and Δ*wecA*Δ*rmpD* were 492 and 162 mg, respectively.

### Mild acid hydrolysis of crude CPS.

To remove lipid A, crude CPS samples were hydrolyzed with 1% AcOH containing 0.1% SDS at 100°C until lipid A precipitate was formed (2 to 4 h). The samples were cooled, neutralized with 2.5 M NaOH to pH 6, and centrifuged at 13,000 × *g* for 20 min. The carbohydrate-containing supernatant was dialyzed against 10 mM Tris-HCl buffer (pH 8), 0.2 M NaCl, and 5 mM EDTA and then against water using Spectra/Por 6- to 8-kDa MWCO dialysis tubing.

### Sonication and *O*-deacetylation of AcOH-treated CPS.

To reduce the molecular weight of polysaccharides for NMR analyses, 20 to 24 mg of AcOH-treated CPS was dissolved in 4 mL water. Samples were sonicated (Fisher Model 500 sonic dismembrator with a microtip) for 20 min at 30% amplitude (10-s on/15-s off) in an ice-water bath. The polysaccharide samples were then purified to remove residual traces of dialysis buffer by chromatography on a Sephadex G-50 superfine column (2.5 cm by 75 cm) and eluted in 50 mM pyridinium acetate buffer (pH 4.5) at a flow rate of 0.6 mL/min. Elution was monitored using a Smartline 2300 refractive index detector (Knauer). The AcOH-treated and sonicated CPS^CELL^ sample from KPPR1S Δ*wecA* was chemically *O*-deacylated with 12% aqueous ammonia at room temperature for 16 h.

### NMR spectroscopy.

AcOH-treated CPS samples (~20 mg, with and without sonication) were deuterium exchanged by being lyophilized twice from 99.9% D_2_O solutions. NMR studies were performed at the NMR Centre of the Advanced Analysis Centre at the University of Guelph. ^1^H and ^13^C NMR spectra were obtained at 40°C in 99.96% D_2_O using a Bruker Avance II 600-MHz spectrometer equipped with a cryoprobe. The chemical shifts are referenced to the internal standard sodium 3-trimethylsilylpropanoate-2,2,3,3-*d*_4_ (δ_H_ = 0 ppm, δ_C_ = −1.6 ppm). The CPS repeat unit structure was established using correlation spectroscopy (COSY), total correlation spectroscopy (TOCSY), heteronuclear single quantum coherence (HSQC), HSQC-TOCSY, and heteronuclear multiple bond correlation (HMBC) NMR experiments. Two-dimensional experiments were performed using standard Bruker software.

### Assessment of HMV by the sedimentation assay.

Overnight cultures of E. coli K30 or K. pneumoniae were subcultured 1:100 and 1:50, respectively, in M9-CAA or LB medium and grown for 5 to 6 h. A total of 100 ng/mL ATc was added to induce gene expression from plasmids. Mucoviscosity was determined by measuring the OD_600_ of the culture supernatant following low-speed centrifugation (1,000 × *g* for 5 min) as described previously (32). All strains that generated a tight cell pellet were string test negative, while those that had turbid supernatants were string test positive. The OD_600_ values of the supernatants were determined for three biological replicates of each strain. Where indicated, samples from these cultures were also collected for analysis by SDS-PAGE and immunoblotting or uronic acid quantification (each described below).

### Capsule SDS-PAGE and immunoblotting.

To examine CPS, bacterial cells (OD_600_ of 1) were collected by centrifugation at 13,000 × *g* for 20 min, and both supernatant and cell pellets were lyophilized. Dried samples were solubilized in 100 μL SDS-PAGE loading buffer, incubated at 100°C for 10 min, and then treated with proteinase K for 1 h (or overnight) at 55°C. SDS-PAGE was performed with 8% acrylamide resolving gels in Tris-glycine buffer (300 V, 3 h, and 4°C). The CPS was stained with 0.25% alcian blue solution in 2% AcOH for 30 min and destained in fixing solution (30% ethanol:10% acetic acid) overnight. The CPS was then stained using the Pierce silver stain kit (Thermo Scientific) (81). For immunoblot analyses, material separated by SDS-PAGE was transferred to Biodyne B nylon membrane (350 mA for 45 min). E. coli K30 CPS was detected using polyclonal antibodies raised in rabbits (1:2,000) (82). Alkaline phosphatase-conjugated goat α-rabbit (1:3,000) was used as a secondary antibody. Immunoblots were developed with nitro blue tetrazolium and 5-bromo-4-chloro-3-indoyl phosphate (Roche Applied Science).

### Bacterial two hybrid assay.

BTH101 cells were transformed with pairs of plasmids with fusions to the two adenylate cyclase domains, with one containing a T18 domain and the other a T25 domain. Controls included in each assay were plasmids encoding only the AC domains (vector control) and a positive control with leucine zipper-AC fusions (47). Colonies were selected on MacConkey agar plates containing 1% maltose, 200 μg/mL carbenicillin, and 50 μg/mL kanamycin. After 3 days, when the color change from interacting pairs was apparent, colonies were subjected to β-galactosidase assays. Cultures grown overnight at 26°C were subcultured into fresh LB broth, incubated at 37°C for 30 min, and then assayed as described (83, 84).

### Wzc and RmpD copurification and immunoblot.

Overnight cultures of E. coli BL21(DE3) with His_6_-Wzc (pLPT045 or pLPT052; Kp) or E. coli BL21 with Wzc-His_6_ (pBRC901 or pBN008; Ec) and RmpD-FLAG_2_ (pKW197) were subcultured 1:100 into 500 mL LB and grown at 37°C to an OD of ~0.1. Then, 100 ng/mL ATc and 10 μM IPTG (pLPT045 and pLPT052) or 0.01% arabinose (pBRC901 and pBN008) were added to induce expression. At an OD of ~1.0, the cells were harvested by centrifugation at 10,000 × *g* for 15 min at 4°C and frozen at −80°C. Thawed pellets were immediately resuspended in lysis buffer (purification buffer [PB] made up of 20 mM NaPO_4_ [pH 7.0], and 0.5 M NaCl with EDTA-free protease inhibitor [ThermoFisher; no. A32955]) and lysed with an EmulsiFlex-C3 high-pressure homogenizer (Avestin, Inc.). Unbroken cells and debris were removed by centrifugation at 12,000 × *g* for 30 min. The supernatant was then centrifuged at 100,000 × *g* for 1 h. The supernatant was discarded, and the pellet containing inner membrane components was resuspended in lysis buffer with 1% *N*-dodecyl-β-d-maltoside (DDM; ThermoFisher; 89902) and incubated at 4°C overnight. The sample was again centrifuged at 100,000 × *g* for 1 h, and the pellet was discarded. The supernatant was incubated with 2 mL of column volume Ni-NTA agarose (previously washed with PB + 10 mM imidazole-0.008% DDM) for 2 h at 4°C with gentle shaking and then packed in a column. The Ni-NTA resin was washed once with 10 mL PB + 10 mM imidazole-0.008% DDM, then twice with 10 mL PB + 20 mM imidazole-0.008% DDM, and twice with 10 mL PB + 35 mM imidazole-0.008% DDM. Proteins were eluted five times with 1 mL PB + 500 mM imidazole-0.008% DDM. All samples were stored at −80°C. SDS-PAGE was performed with 4 to 20% gradient gels (K. pneumoniae) or 15% gels (E. coli) and then transferred to nitrocellulose membranes for immunoblotting. For K. pneumoniae Wzc, mouse α-His (THE His antibody; Genescript) and rabbit α-FLAG (Sigma; no. PA1-984B), both at 1:2,000, were incubated overnight at 4°C following 1-h blocking in 1× phosphate-buffered saline (PBS) with 5% skim milk at room temperature. Fluorescently labeled secondary antibodies (1:25,000; goat α-mouse [no. 926-68070] and goat α-rabbit [no. 926-32211]) in PBS with 5% skim milk were incubated for 1 h at room temperature. Washes performed before and after secondary antibody incubation were done three times for 5 to 10 min each with PBS. Detection was carried out on an Odyssey DLX imager (LI-Cor Biosciences). For E. coli Wzc, mouse α-His (pentaHis; Qiagen; number 34660) and mouse α-FLAG (Sigma; F1804), both at 1:3,000, were incubated overnight in Tris-buffered saline with 1% Tween 20 (TBST) with 3% bovine serum albumin (BSA), following 1-h blocking in TBST + 3% BSA. A horseradish peroxidase (HRP)-labeled secondary antibody (1:5,000; goat α-mouse; Ceaderlane; 115-036-003) in TBST with 5% skim milk was incubated for 1 h at room temperature. Washes performed before and after secondary antibody incubation were done three times for 5 to 10 min each with TBST. Detection reagent (Immobilon Crescendo Western HRP substrate) was applied, and blots were imaged with a Bio-Rad ChemiDoc system. As a control, samples with RmpD-FLAG_2_ and the vector used for the His_6_-Wzc constructs were also analyzed (Fig. S5).

### Assessment of CPS production by uronic acid quantification.

The UA content was measured essentially as described (80, 85, 86). UA was extracted from a 500-μL culture with Zwittergent, precipitated with ethanol, and resuspended in tetraborate-sulfuric acid. Following the addition of phenylphenol, absorbance at 520 nm was measured. UA amounts were determined from a standard curve generated with glucuronolactone.

### Adherence assay.

Adherence assays were performed essentially as described previously (33) using J774A.1 cells. Cells were seeded at 5 × 10^5^ cells per well in a 24-well plate at 16 h before inoculation. At 1 h prior to inoculation, the cells were treated with cytochalasin D to prevent the internalization of the bacteria. Bacteria (~5 × 10^7^) were incubated with J774A.1 cells for 1 h. The samples were rinsed three times with PBS, lysed with 0.1% saponin, and then serially diluted and plated onto LB agar. Recovered CFUs are reported as a percentage of the inoculum CFU.

### Statistics and replicates.

For BACTH and HMV assays, a minimum of three assays were performed, each with three biological replicates. One-way analysis of variance (ANOVA) with Dunnett’s posttest was performed for each data set using GraphPad Prism v9. Typically, a representative experiment is presented. In all bar graphs, bars represent the average values and error bars indicate standard deviations (*n* ≥ 3).
