# Supplementary figures and images for: Hypermucoviscosity Regulator RmpD Interacts with Wzc and Controls Capsular Polysaccharide Chain Length
Source: mBio. 2023 May 4;14(3):e00800-23. doi: 10.1128/mbio.00800-23 (PMC10294653; doi:10.1128/mbio.00800-23)

## A. CPS purification work flow

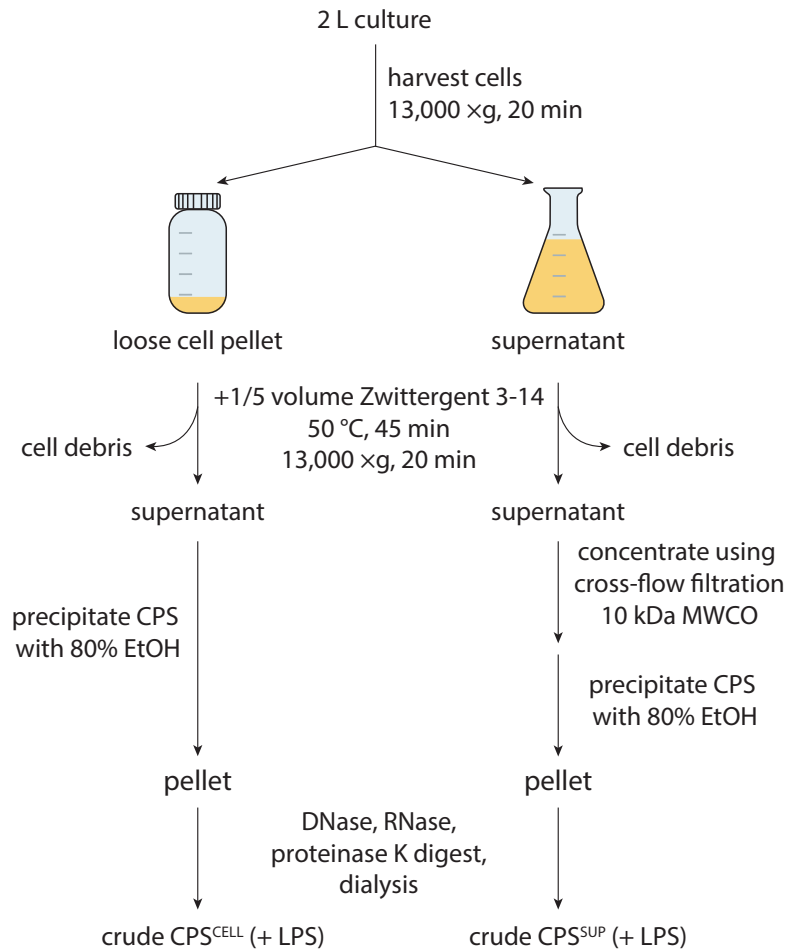

## B. Capsule SDS-PAGE

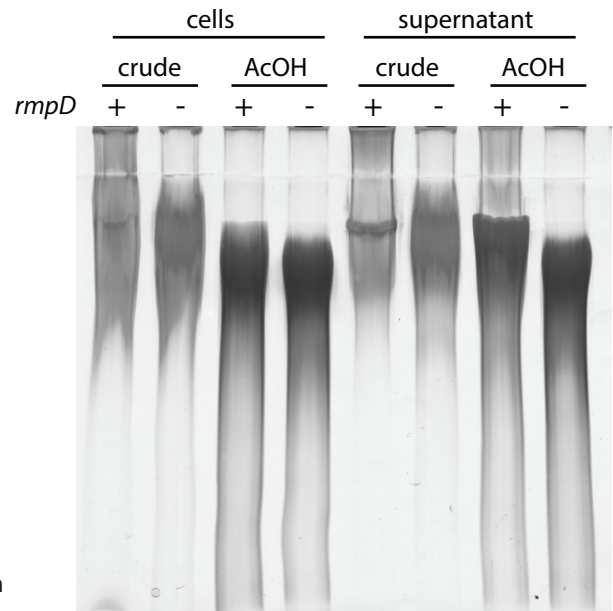

Supplement: FIG S1 [file mbio.00800-23-s0001.pdf]

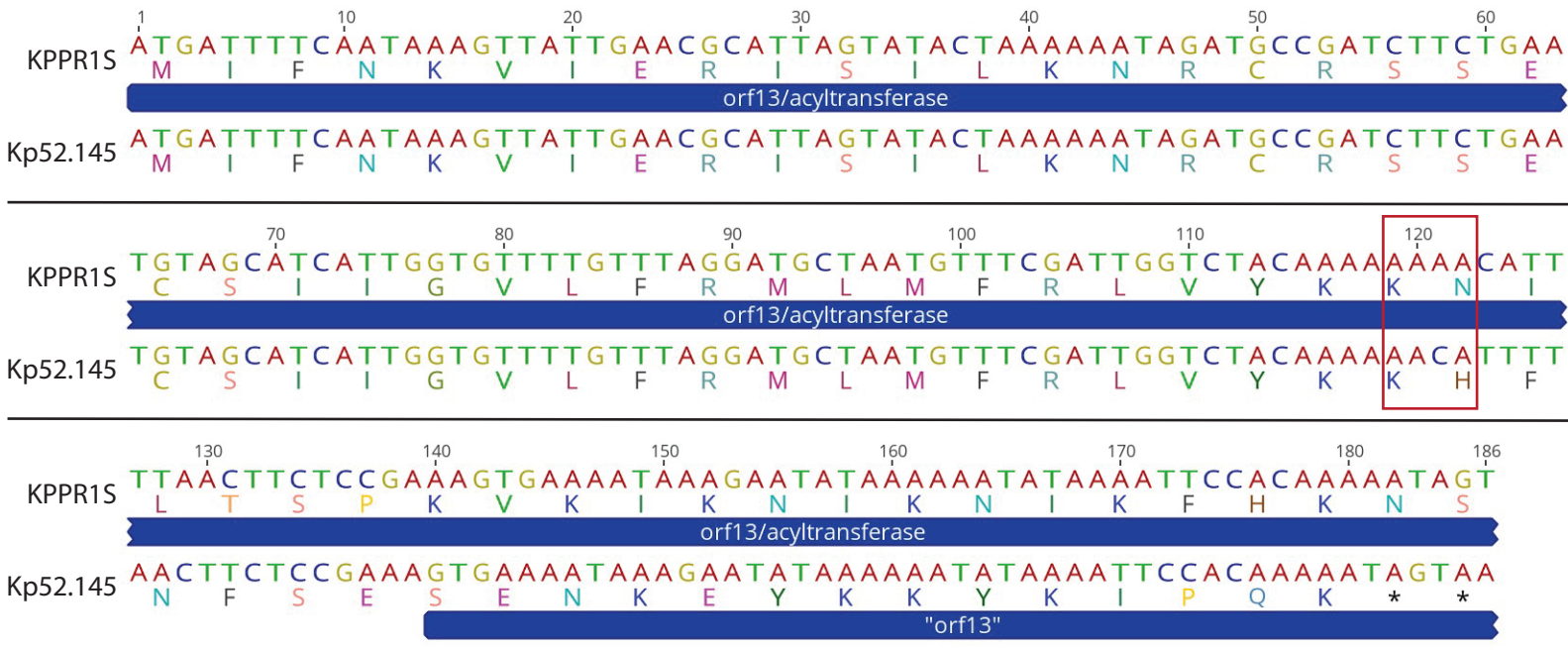

Supplement: FIG S2 [file mbio.00800-23-s0002.pdf]

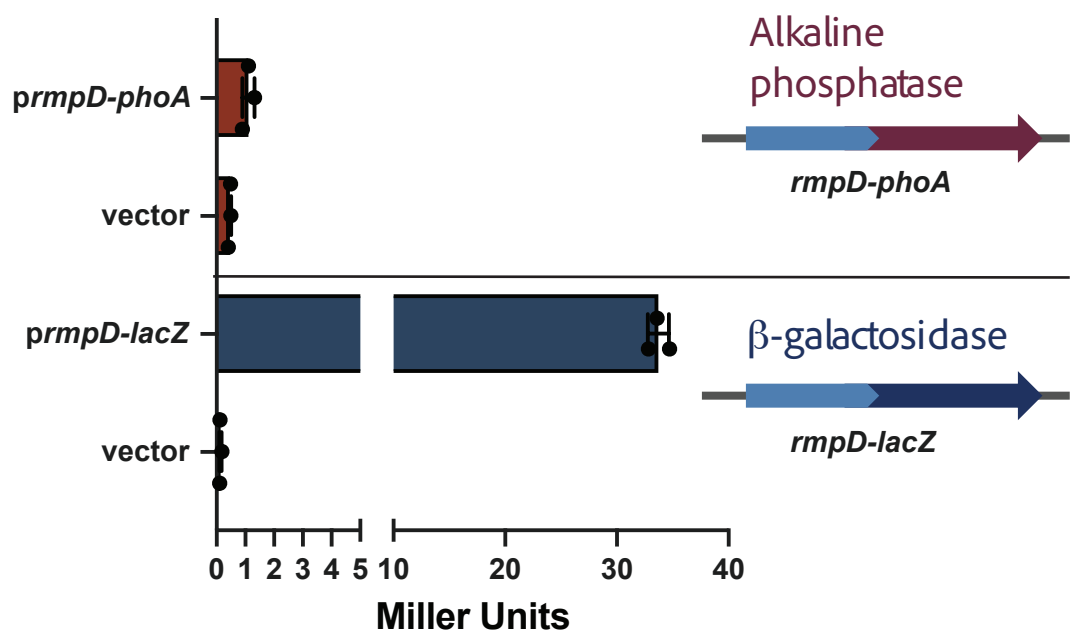

Supplement: FIG S3 [file mbio.00800-23-s0003.pdf]

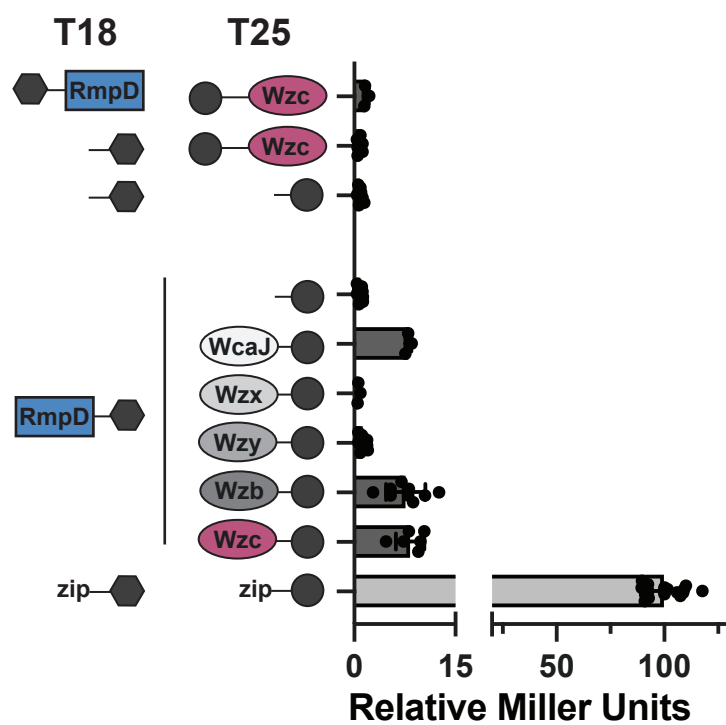

Supplement: FIG S4 [file mbio.00800-23-s0004.pdf]

### A. Uronic Acid

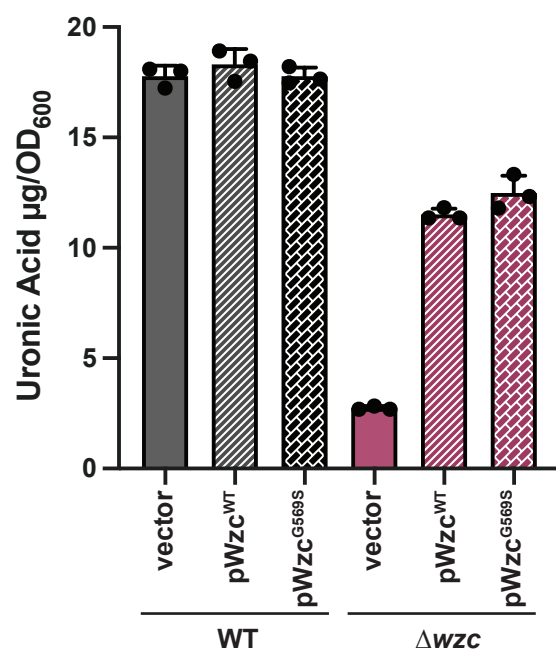

### B. Mucoviscosity

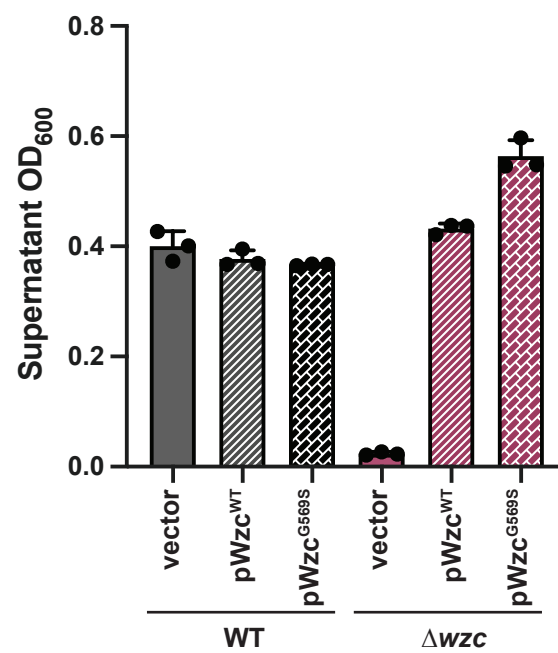

Supplement: FIG S6 [file mbio.00800-23-s0006.pdf]
