# Supplementary material for: Hypermucoviscosity Regulator RmpD Interacts with Wzc and Controls Capsular Polysaccharide Chain Length
Source: mBio. 2023 May 4;14(3):e00800-23. doi: 10.1128/mbio.00800-23 (PMC10294653; doi:10.1128/mbio.00800-23)

A. pRmpD-FLAG$_2$ + pET28a

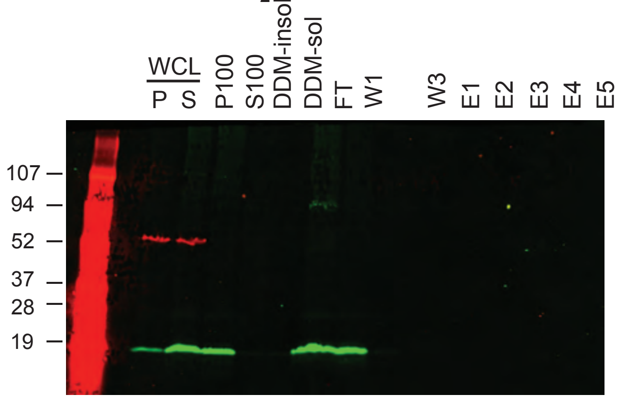

α-His = red α-FLAG = green

B. pRmpD-FLAG$_2$ only

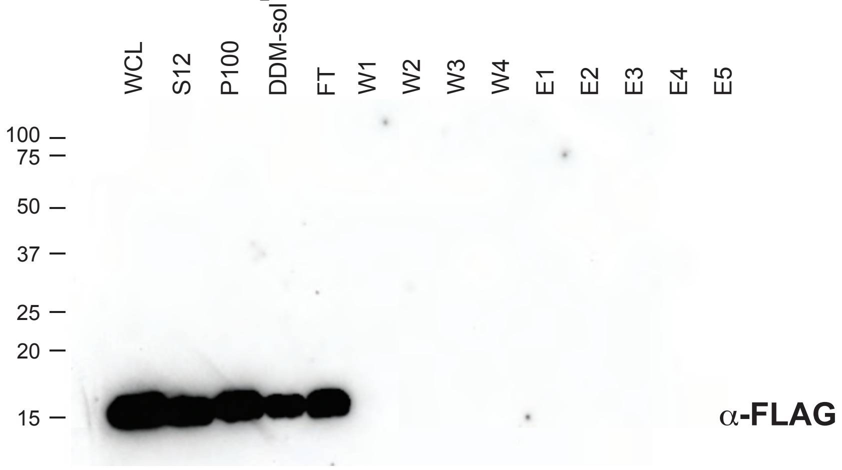

C. pRmpD-FLAG$_2$ + pHis$_6$-Wzc^{WT} (*K. pneumoniae*)

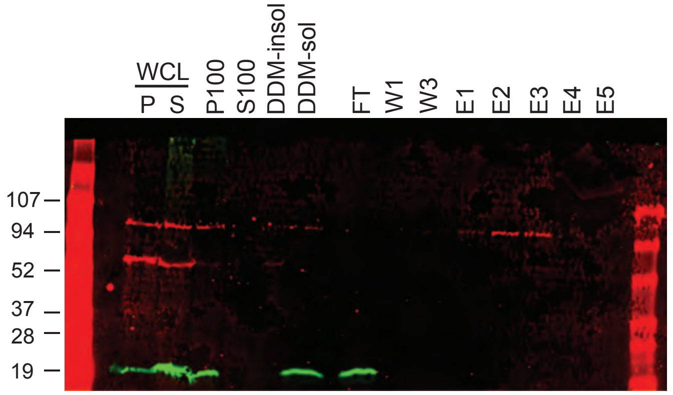

D. pRmpD-FLAG$_2$ + pWzc^{WT}-His$_6$ (*E. coli*)

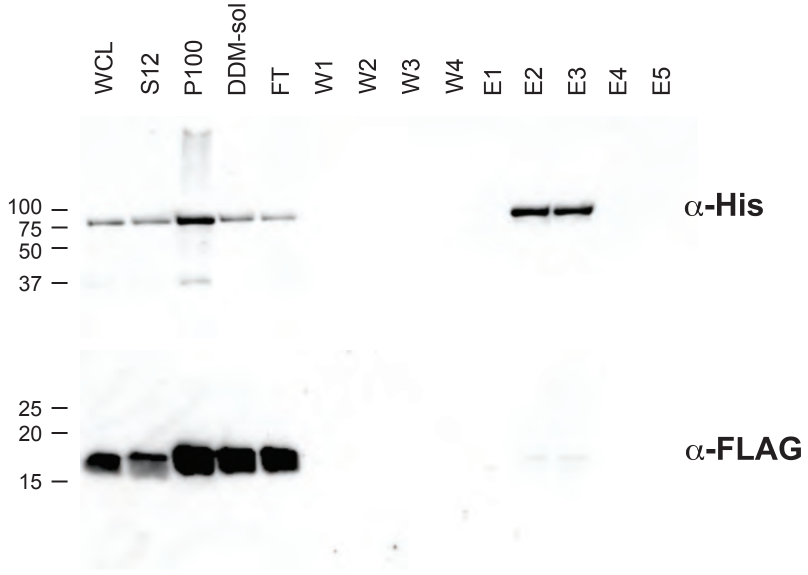

E. pRmpD-FLAG$_2$ + pHis$_6$-Wzc^{K541M} (*K. pneumoniae*)
Dual color image of same blot in Fig 5A

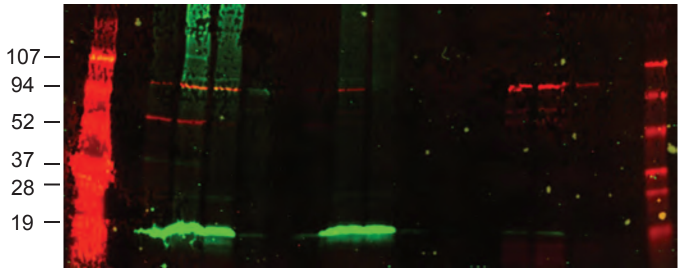

Supplement: FIG S5 [file mbio.00800-23-s0005.pdf]
