# Supplementary material for: Hypermucoviscosity Regulator RmpD Interacts with Wzc and Controls Capsular Polysaccharide Chain Length
Source: mBio. 2023 May 4;14(3):e00800-23. doi: 10.1128/mbio.00800-23 (PMC10294653; doi:10.1128/mbio.00800-23)
Supplement: TABLE S1 [file mbio.00800-23-s0007.pdf]

Table S1. Primers and synthetic genes used in this work

Name	Sequence[a](5'→3')	Use[b]
wecA-delA	GATCTGCGCGCGATCGATATCATGGACGAAGTTATCTACGAAG	F pLPT034 5' flank
wecA-delB	GGTTGAGTCATCACATCCCCAGCTCAACGCATGTGCTCTCAGCATAG	R pLPT034 5' flank
wecA-delC	GAGCTGGGGATGTGATGACTC	F pLPT034 3' flank
wecA-delD	GCGCCAGCTGCAGGCGGCCGCGACGCAACAGATTATTGGCATCC	R pLPT034 3' flank
KW425	GACCATGATTACGCCAAGCTT**C**GTGAAAGCACTCTTTC	F pLPT015
KW426	ACCCGGGGATCCTCTAGA**CG**TTTATTGAATGTGATATATTTTATTAAG	R pLPT015
KW427	GCAGTGGAACGCCACTGCAG**A**AGTGAAAGCACTCTTTC	F pLPT023
KW428	AGCTCGGTACCCGGGGATCCTCATTTATTGAATGTG	R pLPT023
KW429	CCAAGCTTGCATGCCTGCAG**C**ATGACTTCAATATCCAAAAAG	F pLPT014
KW430	AGCTCGGTACCCGGGGATCC**AG**TTTTTTATCTGAATATG	R pLPT014
KW431	GCGCACGCGGCGGGCTGCAG**CT**ATGACTTCAATATCCAAAAAGAAGC	F pLPT013
KW432	ACTTAGGTACCCGGGGATCCCTATTTTTTATCTGAATATG	R pLPT013
KW433	CCAAGCTTGCATGCCTGCAG**C**ATGCATAATATAATTAG	F pLPT012
KW434	AGCTCGGTACCCGGGGATCC**GA**GTTGATGTCATTTTCGGCC	R pLPT012
KW435	GCGCACGCGGCGGGCTGCAG**CT**ATGCATAATATAATTAG	F pLPT010
KW436	ACTTAGGTACCCGGGGATCCTTAGTTGATGTCATTTTCGGC	R pLPT010
KW451	GCGCACGCGGCGGGCTGCAG**CT**ATGTTTAGTACAATATTAATTGTTTG	F pLPT020
KW452	ACTTAGGTACCCGGGGATCCCTAAGCTTTTAATTTCTCAGCC	R pLPT020
KW453	CCAAGCTTGCATGCCTGCAG**C**ATGTTTAGTACAATATTAATTGTTTG	F pLPT019
KW454	AGCTCGGTACCCGGGGATCC**AG**AGCTTTTAATTTCTCAGCCCATAAC	R pLPT019
KW464	TAAAATATATCACATTCAATAAACCTGTTCTGGAAAACCGGGCTGC	F pKW202
KW465	GCTCCACCGCGGTGGCGGCCGCTTATTTCAGCCCCAGAGCGG	R pKW202
KW466	TAAAATATATCACATTCAATAAAGTCGTTTTACAACGTCGTG	F pKW201
KW467	GCTCCACCGCGGTGGCGGCCGCTTATTTTTGACACCAGACCAACTG	F pKW201
KW472	GTATAA**CT**TTTCCTTAAATCAGC	F pKW204
KW473	GCTGATTTAAGGAAA**AG**TTATACTCAC	R pKW204
LT046	TCCTGCAGCCCGGGGGATCCGAGAAATTAAAAGCTTAGAAATTC	F pLPT033/pKW204
LT047	TCCACCGCGGTGGCGGCCGCTTCGTCCCCTTCTTGAGTAAC	R pLPT033/pKW204
LT071	TGCCGCGCGGCAGCCATATGACTTCAATATCCAAAAAGAAGCAACC	F pLPT045/052
LT072	TGTCGACGGAGCTCGAATTCTTCGTCCCCTTCTTGAGTAAC	R pLPT045/052
LT076	CCAAGCTTGCATGCCTGCAG**C**ATGACAATATCTCAGCATCG	F pLPT048
LT077	AGCTCGGTACCCGGGGATCC**AG**ATAAGCAGATTTGTTAATAAATCC	R pLPT048
LT078	GCGCACGCGGCGGGCTGCAG**CT**ATGACAATATCTCAGCATCG	F pLPT047
LT079	ACTTAGGTACCCGGGGATCCTCAATAAGCAGATTTGTTAATAAATCC	R pLPT047
LT087	GCGCACGCGGCGGGCTGCAG**CT**ATGAGTTTAAAATTAAAAACGATAAAAAGC	F pLPT057
LT088	ACTTAGGTACCCGGGGATCCTTATCTCTTTAAGAGCAATTGACG	R pLPT057
LT089	CCAAGCTTGCATGCCTGCAG**C**ATGAGTTTAAAATTAAAAACGATAAAAAGC	F pLPT062
LT090	AGCTCGGTACCCGGGGATCC**AG**TCTCTTTAAGAGCAATTGACG	R pLPT062
LT075	CAAGTCCTAATGCTGGTA**TG**ACATTTGTGAGTTCTAAC	F pLPT052
OL300-F	GATCAAGCTTTCATTTATTGAATGTGATATATT	F pOL300
OL300-R	GATCGGATCCGGTTGATGAAAG	R pOL300

gBlock for pKW201 and pKW202

TTCCTGCAGCCCGGGGGATCCGGTTGATGAAAGATGTCTCATGCTAGGTATTTAGAAAAAAAGGGGAGGAGGGGGTGAAAGCA
CTCTTTCATTTATTTATTTTTTTATTTCTGTTTTATATATCAGTTTATTGTTTTTATTCATATGTGTCAGATAGAAGAAGAATAAAAAA
AATATTCCGCTCGCACAGAACATTGATAAATAGACGAAAAAAATCAAACTTAATAAAATATATCACATTCAATAAA

Primers for *wzc* deletion by lambda red recombinase

LT026 (P1)

GAGCAATCTGCTCTGTTATGGGCTGAGAAATTAAAAGCTTAGAAATTCAGGAAATAATGC*GTGTAGGCTGGAGCTGCTTC*

LT027 (P2)

CATTATTATCCTTTTATTATATATTTTAAAAAAGGGGATTCTTCGTCCCCTTCTTGAGTAAC*ATGGGAATTAGCCATGGTCC*

[a] Blue nt are overhangs for Gibson cloning. Underlined nt indicate restriction sites. Blue bold nt were added to maintain proper coding sequence in BACTH clones. Black bold nt are substitutions to introduce amino acid changes. Italicized nt are the P1 and P2 sequences for lambda red mutagenesis.

[b] F, forward primer; R, reverse primer
